# Study on boiling heat transfer characteristics of R410A outside horizontal tube under swaying condition

**DOI:** 10.1038/s41598-024-52568-5

**Published:** 2024-01-23

**Authors:** Suyu Fei, Haitao Jiang, Dan Hua

**Affiliations:** Jiangsu Key Laboratory of Micro and Nano Heat Fluid Flow Technology and Energy Application, School of Environmental Science and Engineering, Suzhou University of Science and Technology, Suzhou, 215009 Jiangsu China

**Keywords:** Engineering, Statistical physics, thermodynamics and nonlinear dynamics, Thermodynamics

## Abstract

To investigate the boiling characteristics of flow outside the R410A tube under swaying conditions, this article conducts numerical simulation and experimental research on the flow boiling heat transfer of R410A outside a horizontal tube. The results show that when the swing frequency increased from 0.2 to 2 Hz, the sway amplitude is 0.03 m, the heat flux on the inner wall of the runner remains unchanged, and the mass flow rate increases from 85 to 170 kg/(m^2^·s), which makes the heat transfer coefficient of the working fluid in the annular area increases significantly. Keeping the inlet mass flow rate unchanged, the heat flux on the inner wall of the flow channel increases from 25 to 35 kW/m^2^, the heat transfer coefficient of the working fluid in the annular area has also improved, but under high heat flux conditions, the working fluid is evaporated and dried, its heat transfer coefficient increases less than in low heat flux conditions. When the sway amplitude increases from 0.02 to 0.07 m, the sway frequency is 0.2 Hz and 2 Hz respectively, and the heat transfer coefficient of the working fluid shows a downward trend as a whole. The studies provide a reference for heat exchanger design suitable for offshore swaying conditions.

## Introduction

Ocean thermal energy is a large, clean and versatile source of green energy. Ocean thermal gradient power generation is one of the main ways of harnessing ocean thermal energy, which has the advantages of being all-weather, clean and renewable^1^. Ocean thermal gradient power generation uses warm seawater at the ocean surface to heat and vaporise low-boiling working fluids to drive turbines to generate electricity. In engineering, the heat transfer evaporation process between warm seawater and low-boiling working fluid needs to be realised with the help of heat exchangers, so the efficiency of ocean thermal gradient power generation is closely related to the efficiency of heat exchangers^2^.

Heat exchangers have received much attention as an essential ocean thermal gradient power generation component. As a kind of heat exchanger, casing heat exchanger has become one of the most common heat exchange equipment in the chemical, energy and petroleum fields due to its compact structure, small space occupation and good heat exchange efficiency^3^. Scholars from various countries have researched the heat transfer characteristics of casing heat exchangers. Tang et al.^4^ studied the heat transfer characteristics of R134A outside the light tube and the reinforced tube, the results show that boiling is available for the flow of the annular area between the outer tube and the 1EHT tube (There are many pits on the surface of the pipe, which can increase the heat transfer area and surface turbulence), when the average vapor quality is 0.35, the heat transfer coefficient of boiling is 11–36% higher than that of smooth tube. When the average vapor quality is 0.5, the heat transfer performance of 1EHT tubes is not as good as that of smooth tubes. In order to promote the development of heat exchanger reinforced tubes, Dong et al.^5^ experimentally studied the flow boiling heat transfer performance of R410A outside the C-type, I-type and P-type reinforced tubes. The results show that the heat transfer characteristics outside the I-type reinforced tube are the best, and the heat transfer coefficient is 2.1–3.9 times that of the smooth tube. However, there are many tiny depressions on the outer surface of the P-type reinforced tube, and the heat transfer performance deteriorates. Gorgy et al.^6^ experimentally analyzed the heat transfer performance of refrigerants R1234ze(E), R1233zd(E), R123, R134a outside smooth copper pipes and threaded reinforced pipes. The results show that when the heat flux density is 10–60 kW/m^2^, the heat transfer performance outside the reinforced tube is 5.5 times that of the light tube and almost 10 times that of the low heat flux density. Deb et al.^7^ studied the flow boiling heat transfer performance of horizontal smooth tubes and micro-finned tubes using R407c refrigerant. Experimental outcomes demonstrated that the heat transfer rate of the microfin tube is superior to the smooth tube. The pressure drop in regard to R407c was found to be augmented than smooth tube according to the experimental results and enhancement was achieved in microfin tube by 68–110%. Li et al.^8^ studied the condensation flow and heat transfer characteristics of dentate finned tubes by combining numerical and experimental methods. The results showed that the circumferential and axial film thicknesses of the dentate-fin tubes increased with increasing fin density. The mechanism of heat-transfer enhancement for dentate-fin tubes was a joint effect of the heat-transfer area and liquid-film thickness.

Due to the depletion of the ozone layer and the greenhouse effect, HCFC refrigerants have been replaced by some HFC refrigerants in the heat exchange area. R410A has been introduced as an environmentally friendly refrigerant. It has a good heat transfer performance, which is of great importance for studying the heat transfer outside the tube of the shell-and-tube heat exchanger^9–12^. Kim et al.^13^ studied the heat transfer coefficient of R410A and R22 outside the light and micro ribbed tube. It was found that the heat transfer coefficient of 7 kinds of micro ribbed tubes was higher than that of the light tube, and the boiling heat transfer coefficient outside the R410A micro-fin tube was about 1.7–2.9 times that of the light tube. Sun et al.^14^ measured the heat transfer coefficient of R410A refrigerant in saturated flow boiling outside herringbone microfin tubes and 1EHT tubes, and compared them with smooth tubes, the experiments show that when the gap in the annular area is 6.35 mm, the heat transfer coefficient increases with the increase of the mass flow rate. When the gap in the annular area becomes smaller to 2.15 mm and the mass flow rate is low, the heat transfer coefficient decreases due to bubble blockage. Li et al.^15^ further explored the influence of outer fin structure on flow boiling heat transfer and used R410A refrigerant to conduct flow boiling heat transfer experiment outside the reinforced tube of three different outer fin structures. Experiments show that the heat transfer performance of R410A outside the reinforced tube of microporous structure is the best, the heat transfer coefficient is about 2.52–2.64 times that of the smooth tube, and the heat transfer coefficient of the measured tube can be accurately predicted by correcting the correlation coefficient within the deviation range of ± 10%. Li et al.^16^ conducted an experimental study on the two-phase heat transfer of refrigerant R410 A in the outer annulus of smooth tube and enhanced tube. The results indicated that: herringbone structure is more effective in condensation, especially at low flow rates, and the dimple structure is more effective in evaporation. DIM/HB tube shows the highest performance factor in two-phase heat transfer.

At present, in the study of heat transfer performance of seawater heat exchanger, the influence of seawater flow on convective heat transfer outside the tube of heat exchanger is usually not fully considered. In fact, the sloshing caused by the flow characteristics of seawater has a very important influence on the heat transfer process of seawater heat exchanger. Du et al.^17^ combined the multi-component model in FLUENT software with the customized multi-component mass transfer model to study the heat and mass transfer characteristics of the shell side of the vapor–liquid two-phase mixed refrigerant in the spiral wound heat exchanger. It is found that the sloshing condition improves the heat transfer performance, but sometimes the effect is not significant. The sloshing condition is beneficial to reduce the flow resistance. Hu et al.^18^ studied the flow boiling heat transfer characteristics of printed circuit heat exchangers (PCHEs) under different oscillation conditions under the regulation of a 6-degree-of-freedom motion platform. The results show that, among the six sloshing types, rolling and heaving movements have significant effects. Rolling conditions always deteriorate the flow boiling heat transfer in PCHEs. Heaving conditions always enhance the flow boiling heat transfer in PCHEs. Ren et al.^19^ used ANSYS software to simulate the sloshing motion, and analyzed the influence of sloshing motion on the flow and heat transfer characteristics of the shell side. As a result, the shell-side flow and heat transfer characteristics were obviously influenced by sloshing motions and showed periodicity. The effects of pitching and heaving were more significant than that of rolling, and the effect of sloshing period was smaller than that of sloshing amplitude.

Although considerable progress has been made in the study of the flow boiling heat transfer characteristics of shell-and-tube heat exchangers, most of the studies on the flow boiling heat transfer characteristics of refrigerants remain in the land-based stable conditions, and the research on the flow heat transfer outside the tube suitable for the sloshing conditions of offshore facilities is not seen in the literature. In addition, most of the studies on flow boiling under sloshing conditions also discuss the influence of sloshing type on heat transfer characteristics, and do not discuss the specific influence of frequency and amplitude on flow boiling. Therefore, in this paper, a two-phase flow heat transfer test rig was set up, and R410A was used as the working fluid to study its flow boiling characteristics outside the horizontal pipe. On this basis, the effects of different oscillation frequencies and different oscillation amplitudes on the flow boiling characteristics outside the R410A tube are analysed by adding oscillation excitation, which provides a basis for the design of heat exchangers suitable for offshore sloshing conditions.

## Experimental apparatus and methods

### Experimental system

**Figure 1:**
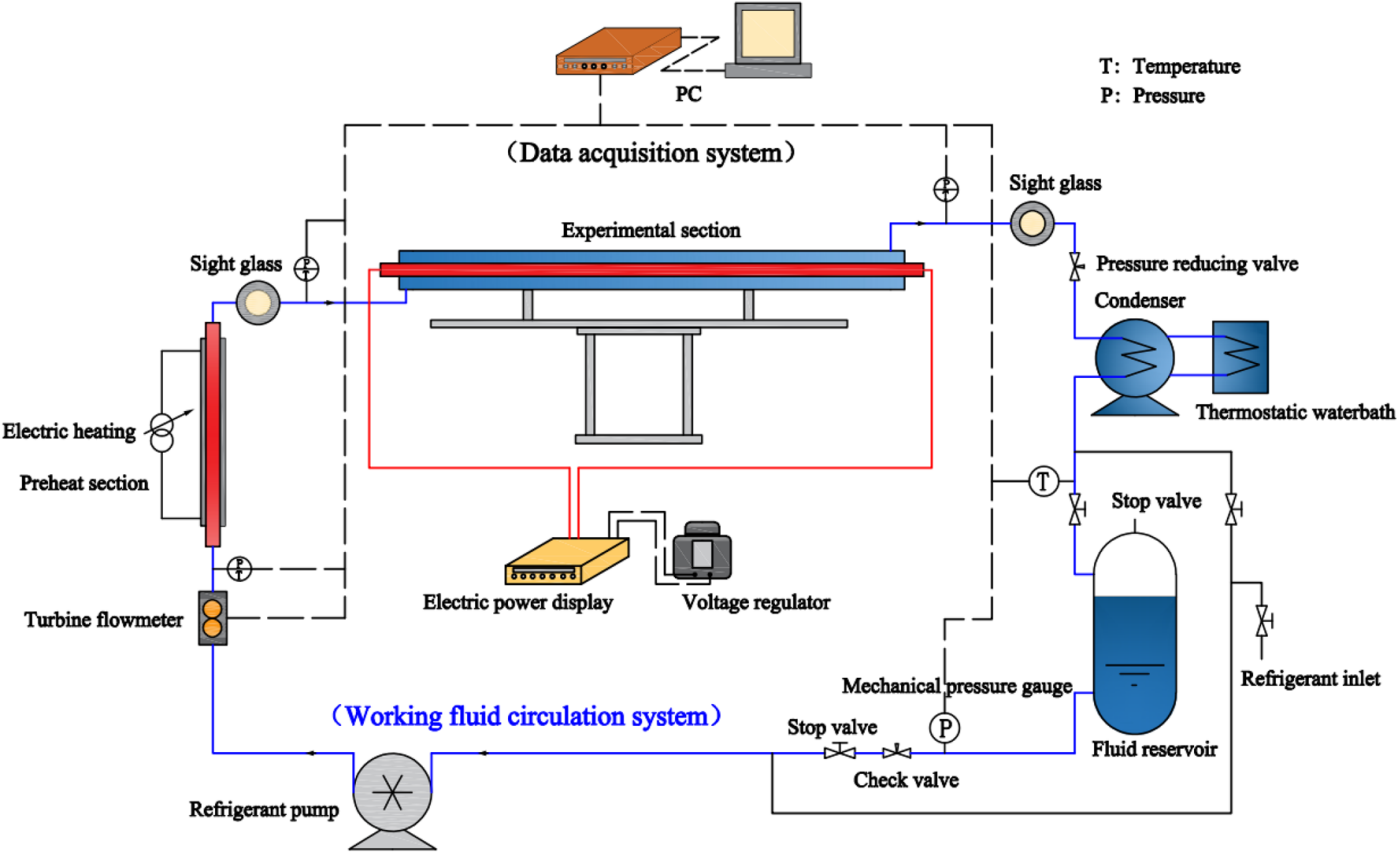
Experimental system schematic.

As shown in Fig. 1, the experimental system mainly includes four parts: a working fluid circulation system, a cooling water circulation system, a heating system and a data acquisition system. The working fluid circulation system is the core part.

**Figure 2:**
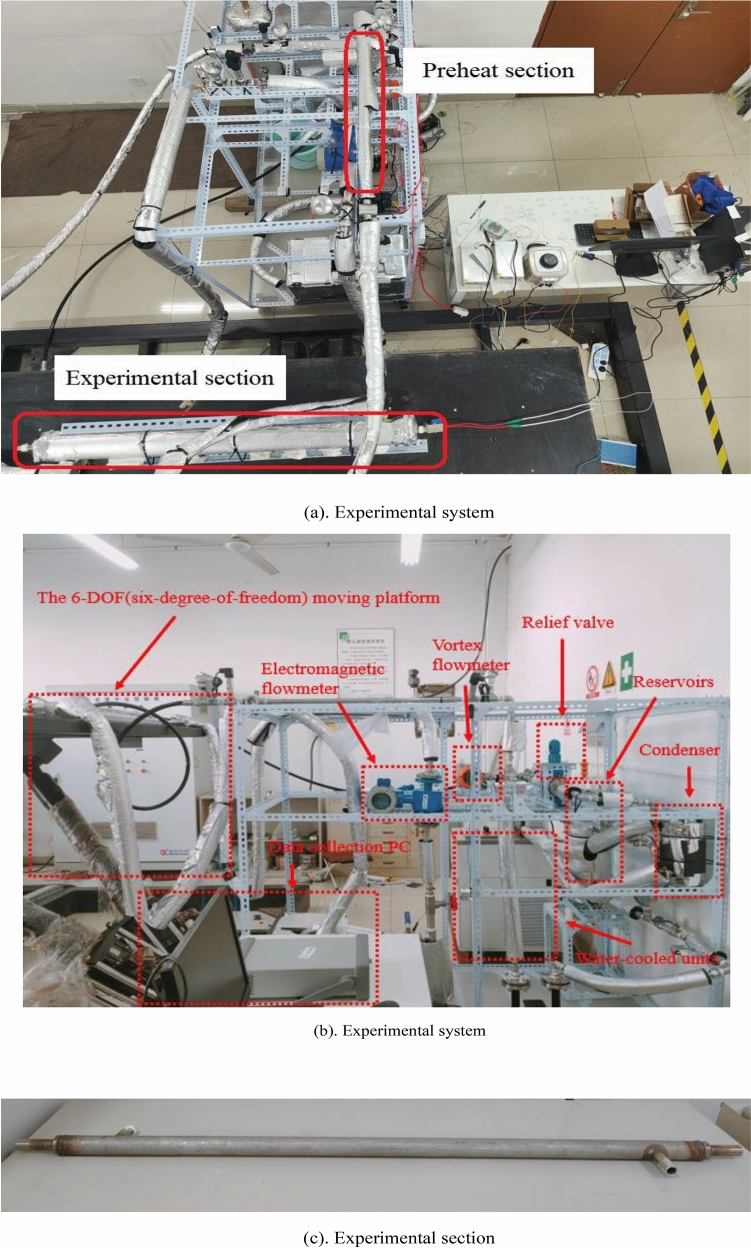
Experimental rig.

The working fluid circulation system mainly composes a refrigerant pump, turbine flowmeter, preheat section, experimental section, sight glass, pressure reducing valve, condenser, fluid reservoir, mechanical pressure gauge, temperature and pressure sensor and valve. Figure 2a,b and c show the experimental system and section.

Working fluid cycle process: firstly, the mass flow rate of the working fluid is controlled by adjusting the refrigerant pump frequency. Subsequently, the working fluid is transported to the preheating section to be heated to the specified inlet state. The preheater function is to adjust the vapor quality and temperature of the working medium and provide an ideal state for the entrance of the experimental section. Through preheater heating, the working fluid reaches saturation at its outlet, and directly absorbs heat and evaporates after entering the experimental section. The experimental section's horizontal casing features an electric heating rod that heats the working fluid. The heated working fluid flows through the channel, absorbing heat and subsequently evaporating and boiling. Sight glasses are equipped before and after the experimental section to allow observation of the working fluid's state. The working fluid passes through the condenser, undergoes condensation, and returns to the liquid storage tank to complete the cycle. The experimental section's heating system comprises a heating rod, a frequency converter voltage regulator and a power display.

### Experimental conditions

In this experiment, as shown in Table 1, the inlet saturation temperature is consistently controlled within the range of 25 ± 0.2 °C. The mass flow rate of R410A varies from 85 to 250 kg/(m^2^·s), and the average heat flux ranges from 25 to 35 kW/m^2^.

**Table 1:** Test parameters of experimental conditions.

Project	Parameter
Working fluid	R410A
Mass velocity (kg/m^2^·s)	85–250
Inlet saturation temperature (°C)	25 ± 0.2
Heat flux (kW/m^2^)	25–35

The installed whole casing is tightly wrapped with thermal insulation material. The single-phase heat transfer experiment verifies that the heat loss does not exceed 5% to ensure the adiabatic boundary condition of the outer wall of the casing. The criterion of the stable state in the experiment is that all the temperature fluctuations in the system are stable between ± 0.2 °C.

**Figure 3:**
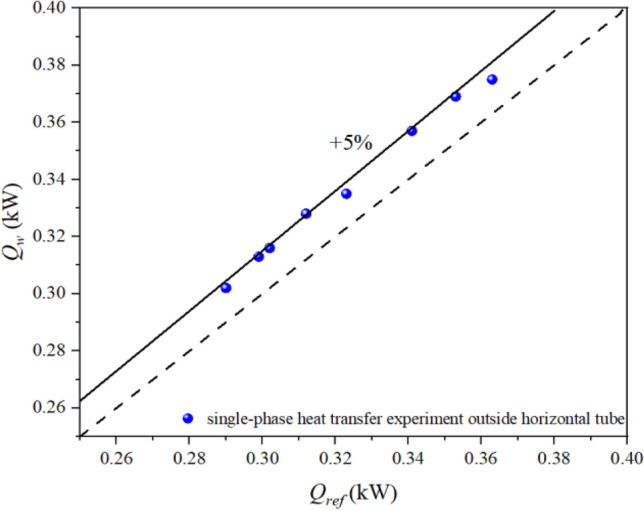
The heat loss of the single-phase experiment outside the tube.

The key parts of the experimental system and the preparation work before the start of the experiment need to be well insulated to achieve the most ideal experimental condition. All exposed parts of the experimental equipment are insulated by wrapping them in insulation wool to minimise heat loss. Although careful thermal insulation has been done, but the experimental section in the refrigerant side and the water side of the heat transfer still exists in heat loss. Therefore, the heat balance needs to be analysed first to ensure that the heat loss is small. As can be seen from the Fig. 3, the heat loss of the single-phase experiment outside the tube is basically within 5% within the range of mass flow rates tested, thus indicating that the experimental section has a good heat balance and a good insulation effect.

### Experimental data processing

The heat leakage of R410A in the experimental section *Q*_*L*_:

1 $$Q_{L} = \frac{{T_{w} - T_{air} }}{{\frac{1}{{A^{\prime}h^{\prime}}}}} + \frac{{T_{w}^{4} - T_{air}^{4} }}{{\frac{1}{{\sigma A^{\prime}}}}}$$

where *h'* is free convection heat transfer coefficient; *A'* is the external surface area of the experimental section; *T*_*w*_ is the outer wall temperature of the steel pipe in the experimental section; *T*_*air*_ is the temperature of ambient air; *σ* is blackbody radiation constant.

The heat rate is determined by Ohm’s law and is given by Q1:

2 $$Q_{1} = UI$$

where *U* is voltage at both ends of the heating rod, V; *I* is the current intensity through the heating rod, A.

The heat flux density q of the heating rod in the experimental section:

3 $$q = \frac{{Q_{1} - Q_{L} }}{2\pi rL}$$

where *r* is the radius of the heating rod in the experimental section; *L* is the length of the experimental section.

Scholars have proposed many based on the correlation of boiling heat transfer coefficient of two-phase flow in the tube. Due to the difference between the flow boiling heat transfer in the tube and the flow boiling heat transfer in the annular region and the relative lack of experimental data. There are relatively few predictive correlations for the annular region's flow boiling heat transfer coefficient. The correlation of Gungor and Winterton^14^ is acknowledged as an assessment of the flow boiling heat transfer coefficient within the annular region.

## Numerical simulation

The above experiments are only carried out for stable working conditions and because the existing experimental platform is not equipped with equipment that simulates the swaying conditions at sea. Therefore, the study of flow boiling heat transfer outside the tube with sway excitation will be analyzed by numerical simulation.

### Mathematical model

#### Turbulent flow model

For turbulence models, numerous selection methods exist within engineering, with the standard k–ε model being the most extensively employed and critical tool in engineering fluid calculation.

#### VOF model

For selection the multiphase flow model, the explicit solution method of the VOF model is used to simulate the temperature distribution, velocity distribution and heat transfer coefficient of gas–liquid two-phase flow outside the tube.

#### Phase transformation model

In studying mathematical phase transition models, Lee's gas–liquid phase transition model^15^ is widely used in engineering applications. This paper also uses the model to realize the phase transition process of R410A outside the horizontal tube.

Boiling process:

4 $$\mathop m\limits^{ \cdot }{_g} = - \mathop m\limits^{ \cdot }{_l} = r^{\prime}\alpha_{l} \rho_{l} \frac{{T - T_{sat} }}{{T_{sat} }},\quad T > T_{sat}$$

Condensing process:

5 $$\mathop m\limits^{ \cdot }{_l} = - \mathop m\limits^{ \cdot }{_g} = r^{\prime}\alpha_{g} \rho_{g} \frac{{T - T_{sat} }}{{T_{sat} }},\quad T > T_{sat}$$

where *α*_*l*_ and *α*_*g*_ are the volume fractions of the liquid phase and gas phase, respectively; *ρ*_*l*_ and *ρ*_*g*_ are the densities of the liquid phase and gas phase, respectively; *T* and *T*_*sat*_ are the temperature and saturation temperature of the working fluid outside the tube, respectively; *r'* is the mass transfer intensity factor.

#### Swaying model

There are many numerical simulation methods for sloshing, such as MPS method^16^, SPH method^17^, UDF method^18^, etc. External transverse excitation is added to R410A at the inlet^18,19^ to simulate its evaporation process under sway conditions. The external transverse excitation given by is:

6 $$y = v + A\sin \left( {2\pi ft} \right)$$

where *v* is the base velocity of inlet working fluid; *A* is the swaying amplitude; *f* is the swaying frequency.

### Physical model

**Figure 4:**
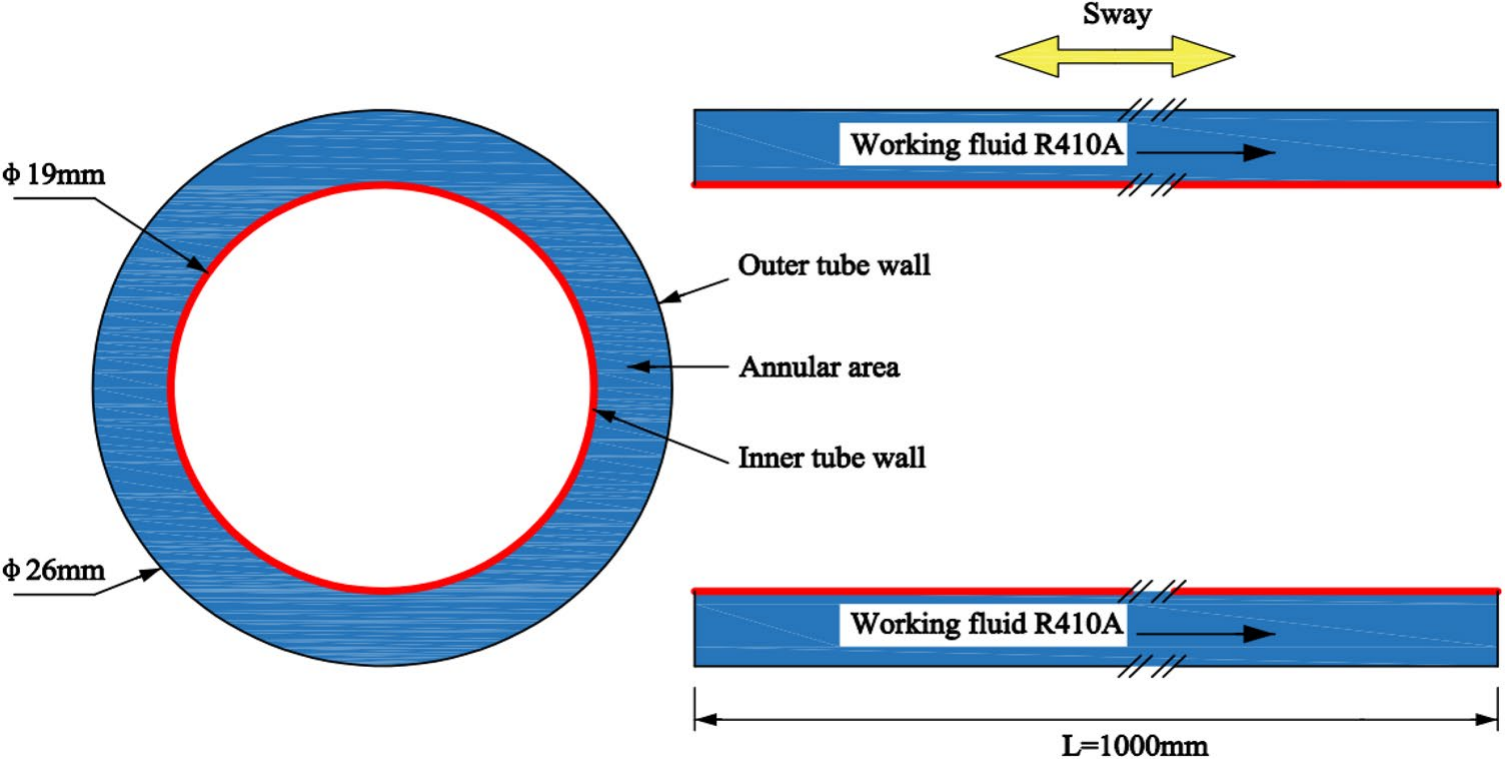
Geometric model of horizontal pipe.

To provide a more intuitive simulation of R410A flow boiling outside the tube, the horizontal casing model used in the above experiment has been simplified. Simulation now only involves the creation of a grid in the annulus area, as shown in Fig. 4. The casing annulus area serves as the working fluid flow channel, with the inner wall surface subject to constant heat flux density. The outer wall of the channel does not exchange heat with the environment, making it an adiabatic boundary. The annular area has an outer diameter of 26 mm and an inner diameter of 19 mm, with a tube length of 1000 mm. To ensure precision and consistency, the horizontal casing utilizes a structured O-type grid.

### Boundary conditions and solution settings

R410A was selected as the working fluid, and its physical parameters were inquired by Refprop software. When the temperature is 25 °C, the density *ρ* = 1058.9 kg/m^3^, the specific heat capacity *c*_*p*_ = 1707.7 J/(kg·K), the thermal conductivity *λ* = 0.0892 W/(m·K), the viscosity *μ* = 0.118 kg/(m·s). In addition to the above governing equations, it is also necessary to determine the boundary conditions and initial conditions of the equation to make the equation's solution unique. The boundary conditions of the control body are shown in Table 2.

**Table 2:** Boundary conditions.

Location	Boundary types	Explanation
Import of R410A	Velocity-inlet	Consideration of flow and pressure constraints in boundary parameters of heat exchange tube test
Export of R410A	OUTFLOW	Export flows are eligible for full development
Inner wall of flow channel	Constant heat flux wall	More suitable for experimental data than constant temperature wall
Outer wall of flow channel	Thermally insulated surface	Satisfy the conditions for outer tube wall wrapping insulation material to achieve heat insulation

After defining the boundary conditions, only the solution needs to be chosen. The relaxation factor and step size for the transient solution must be configured. The initial conditions used in the simulation calculation are presented in Table 3.

**Table 3:** Initial conditions.

Project	Format
Solver	Three-dimensional, unsteady state
Gravity	− 9.81 m/s^2^
Turbulence model	Realizable k–ε model
Multiphase flow model	VOF model
Phase setting	Prime phase: R410A liquid; second phase: R410A gas
Saturation temperature of R410A	25 °C
Surface tension model	CSF
Pressure–velocity coupling algorithm	PISO
Pressure discrete format	PRESTO!
Discrete format of momentum equation	Second Oeder Upwind

### Grid independence verification

**Figure 5:**
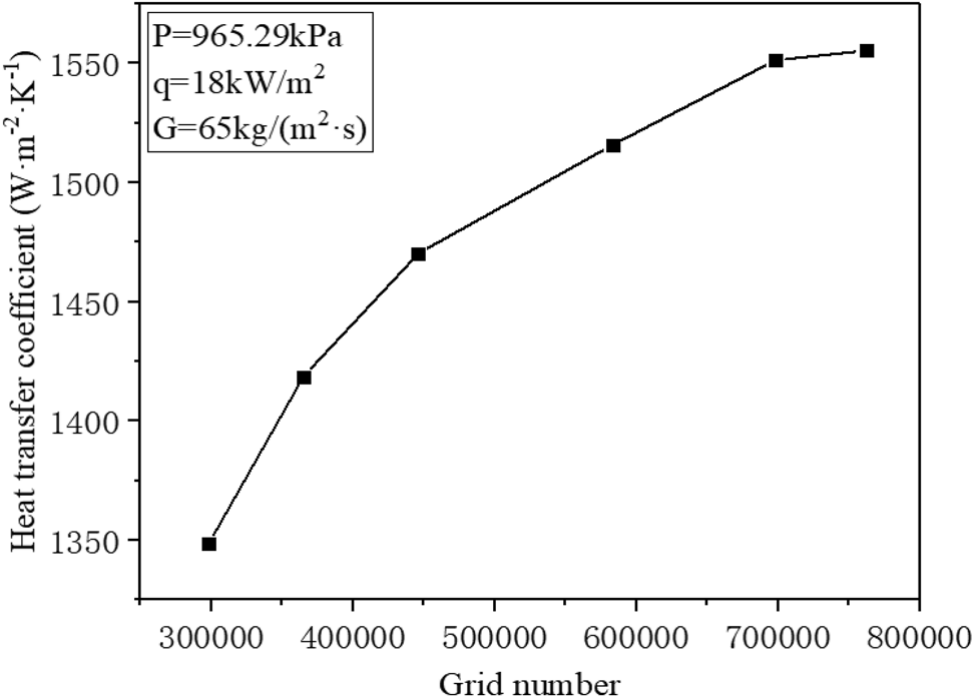
Grid independence verification.

In order to achieve fast, convergent, and stable results, it is essential to verify the grid-independence prior to calculation. Thus, six various grid numbers were selected: 298300; 365400; 446700; 553800; 697800; and 762400. The calculation was performed under the same conditions for each of these grids. When the heat flux reaches 18 kW/m^2^ and the mass flow rate is 65 kg/(m^2^·s), Fig. 5 illustrates the change in the convective heat transfer coefficient outside the R410A tube as a function of the number of grids. The figure indicates that the numerical simulation results are only minimally affected by the number of grids when it exceeds 697800. Therefore, for the sake of calculation accuracy and cost, 697800 grids have been chosen for the model.

**Figure 6:**
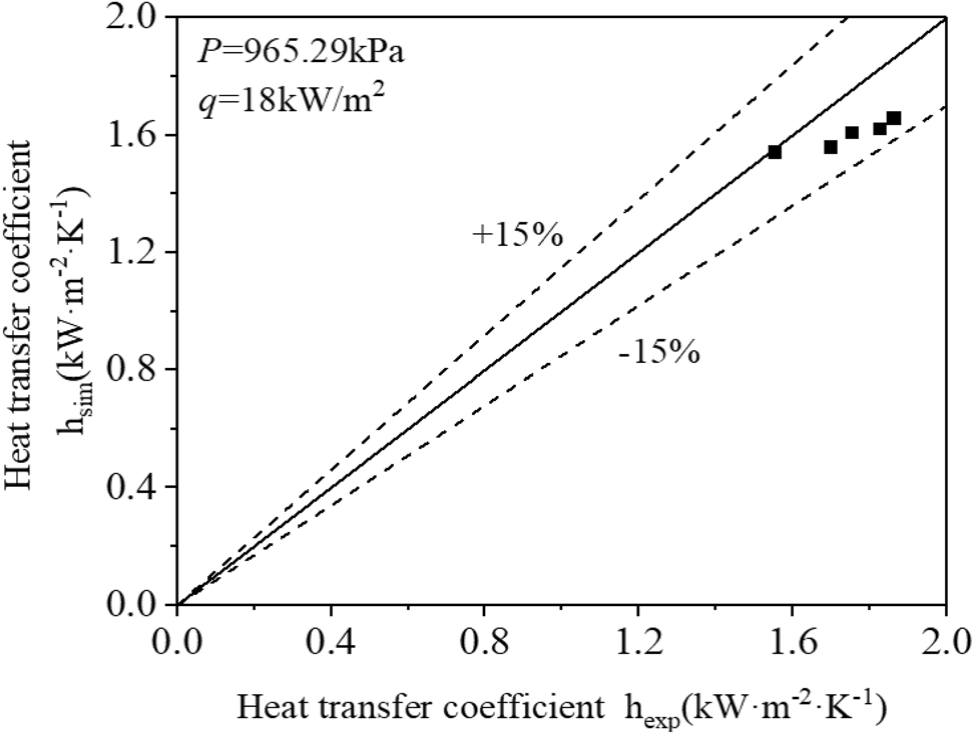
Error analysis of numerical calculation results and experimental results.

To guarantee the precision of the numerical findings, it is crucial to assess the disparity between the numerical simulation outcomes and the experimental results under the equivalent working conditions as depicted in Reference^20^ prior to the formal numerical analysis. Figure 6 displays the outcomes. The flow boiling heat transfer test utilises R410A as the heat transfer agent, with a saturation temperature of 6 °C and a mass flow range of 55–80 kg/(m^2^·s). The findings indicate that, when working under identical conditions, the discrepancy between numerical and experimental outcomes does not exceed 15%, whilst displaying a high correlation. These results thereby authenticate the dependability of both the numerical simulation technique and calculation methodology.

## Results and discussion

### Comparison of experimental and simulation data under stable conditions

**Figure 7:**
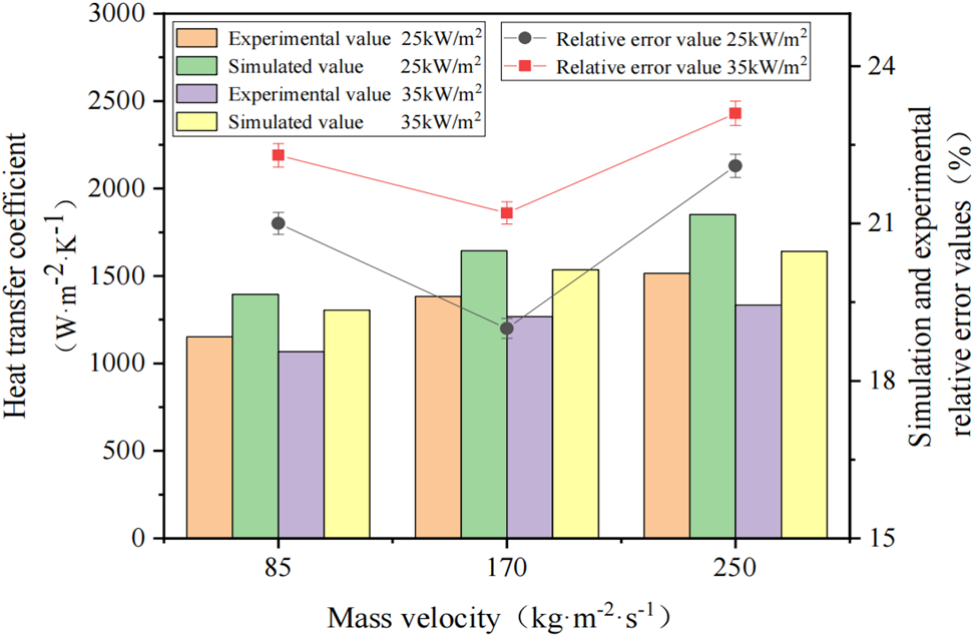
Comparison of experimental and simulated values of heat transfer coefficient under stable conditions.

The inlet mass flow rates of R410A are 85 kg/(m^2^·s), 170 kg/(m^2^·s) and 250 kg/(m^2^·s), and the heat flux densities on the inner wall of the flow channel are 25 kW/m^2^ and 35 kW/m^2^. The heat transfer coefficient of flow boiling outside the R410A horizontal tube under stable working conditions was measured and calculated based on the above experimental and simulation methods. The effects of different mass flow rates and heat flux densities on the heat transfer coefficient under stable conditions, the experimental values and the simulated values are shown in Fig. 7.

Figure 6 illustrates that, under stable working conditions, the experimental value of the flow boiling heat transfer coefficient outside the horizontal tube is lower than the simulated value at the same mass flow rate and heat flux. This can be attributed to the effect of thermal resistance and heat leakage during the experimental process, which reduces the heat transfer coefficient. However, the relative error between the two values is below 25%, and overall, the data's results are within a reasonable range. When the mass flow rate ranges from 85 to 250 kg/(m^2^·s) and the heat flux is between 25 and 35 kW/m^2^, the heat transfer coefficient of R410A in the annular region increases with the increase in mass flow rate under the same heat flux. This is because a higher mass flow rate at the inlet results in a longer liquid phase region of the working fluid, a shorter boiling section length, and a smaller heat transfer thermal resistance formed by the gas phase. At the same time, the disturbance in the fluid domain is intensified, leading to an increase in the heat transfer coefficient. However, at the same mass flow rate, an increase in heat flux leads to a decrease in the rate of increase of the heat transfer coefficient. As the heat flux increases, the overall heat transfer coefficient of R410A increases. However, the length of the boiling section of the working medium in the flow channel is increasing, and the thermal resistance to heat transfer formed by the gas phase is also increasing. This results in a decrease in the heat transfer coefficient under relatively low heat flux.

### Numerical simulation comparison of stable condition and swaying condition

**Figure 8:**
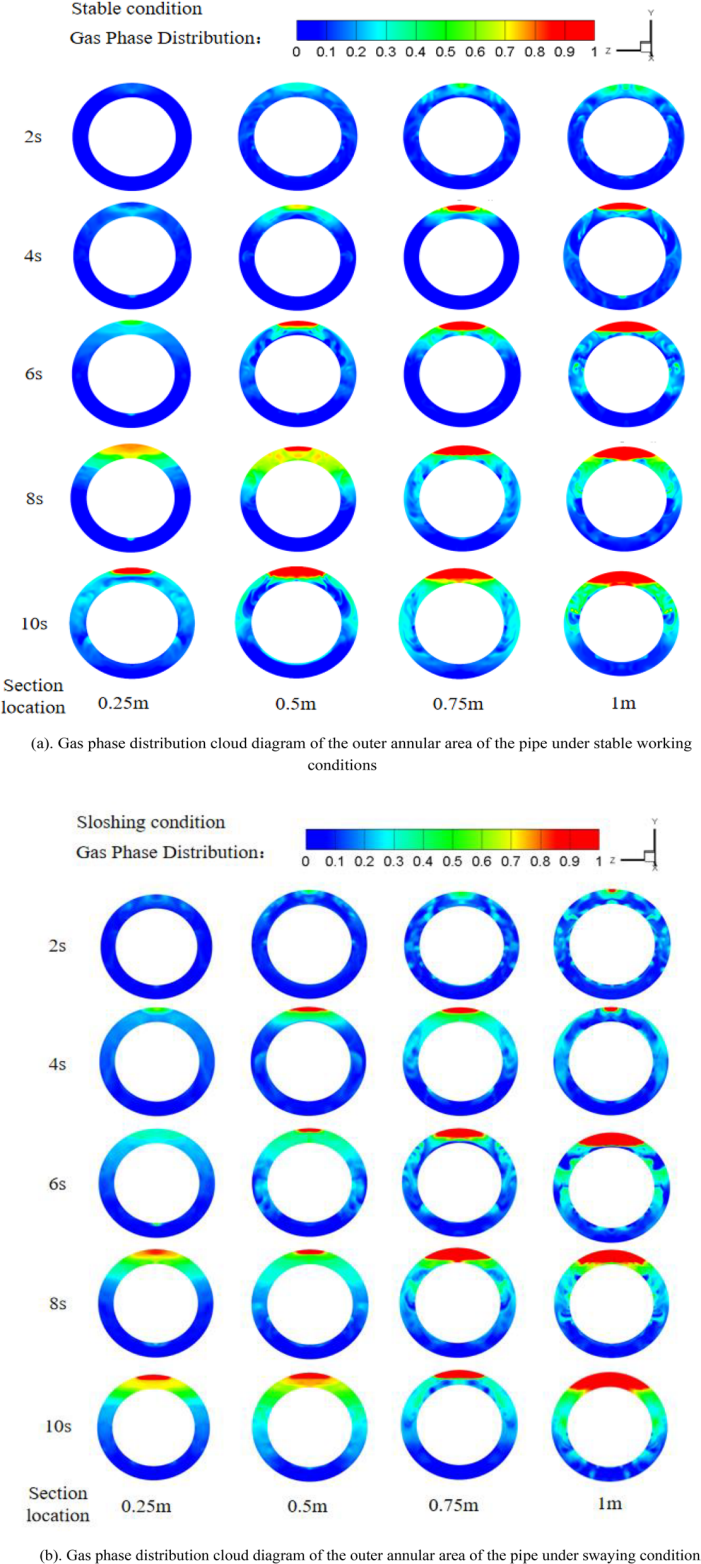
(**a**). Gas phase distribution cloud diagram of the outer annular area of the pipe under stable working conditions. (**b**) Gas phase distribution cloud diagram of the outer annular area of the pipe under swaying condition.

The inlet mass flow rate of R410A is 85 kg/(m^2^·s), and the heat flux density on the inner wall of the channel is 25 kW/m^2^. The steady condition and the condition with the sway frequency of 0.2 Hz and the sway amplitude of 0.03 m are compared. The gas phase distribution at the cross section of the flow direction of the working fluid along the annulus side of the casing during the 10 s time is shown in Fig. 8a and b.

It is evident from the figure above that R410A undergoes phase change as it flows outside the tube. Owing to gravity, the liquid is largely distributed below the annulus area outside the tube, while the gas is predominantly located above the annulus area. The concentration of gas steadily increases along the working medium's flow direction and reaches its peak at the outlet section. This is because, in the early stage of liquid flow, the temperature of the inner wall of the channel is relatively low and hasn't reached the superheat required for bubble formation. As heat continues to flow, the temperature of the channel's inner wall gradually increases, and it reaches the necessary superheat for the inception of bubbles, causing bubbles to form on the wall. When the bubble reaches a certain size, it detaches from the heat exchange tube's wall surface due to the annular side fluid's flow. The fluid in the annulus region flows forward while the wall temperature and vaporization core size increase, resulting in more abundant and faster bubble formation. The mainstream liquid temperature also rises, reducing the level of supercooling and enhancing gas content. From the gas phase distribution cloud map, comparing the swaying and stable conditions, the bubble core appears more prominent and fluid movement intensifies. This occurs because, under the swaying condition, the fluid velocity in the annulus area is constantly changing, which enhances convective heat transfer between the working medium and the inner wall surface, resulting in relatively conspicuous bubble formation.

**Figure 9:**
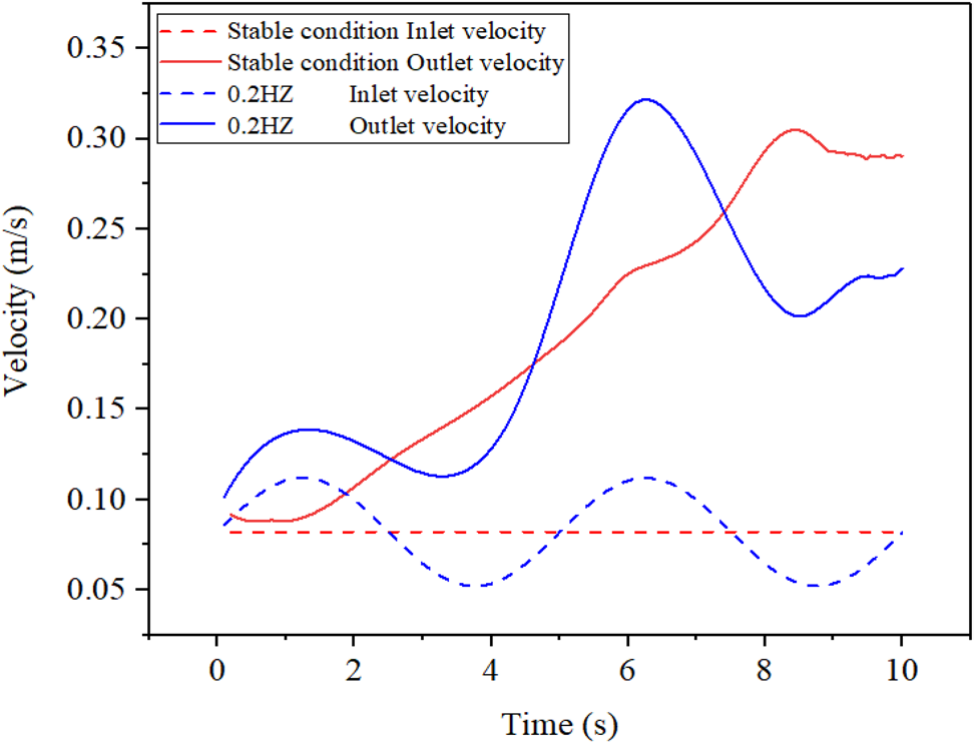
The variation of inlet and outlet velocity with time.

The working condition with a sway frequency of 0.2 Hz and sway amplitude of 0.03 m has been chosen. This allows for a comparison of the inlet and outlet velocity and heat transfer coefficient with the corresponding stable working condition, to investigate the features of both the swaying and stable working states. Figure 9 illustrates the changes in inlet and outlet velocity over time for both swaying and stable conditions. The diagram illustrates that, under stable conditions, the inlet flow rate of R410A remains constant while the outlet flow rate typically increases over time. When subject to 0.2 Hz oscillations, the inlet velocity oscillates sinusoidally, while the outlet velocity generally increases. However, it is important to note that there is a notable decrease in speed from 2 to 4 s and 7 s to 9 s, which is attributed to the change in outlet speed caused by sway excitation.

**Figure 10:**
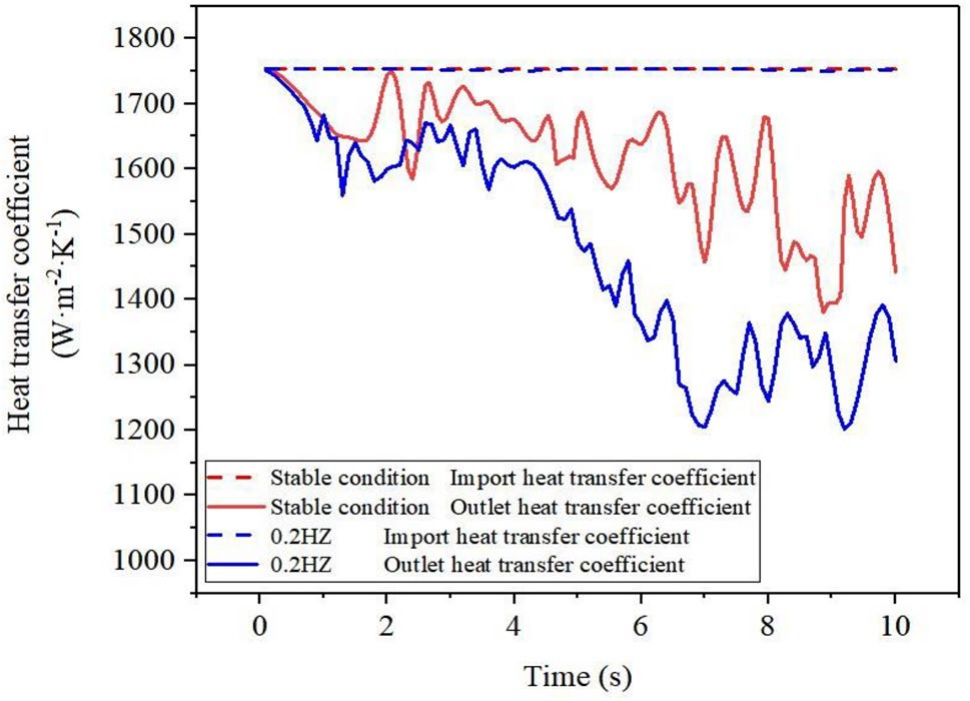
Variation of heat transfer coefficient of inlet and outlet with time.

Figure 10 displays a comparison diagram of the inlet and outlet heat transfer coefficients over time, both under stable and sway conditions. The figure demonstrates that, under stable conditions, the inlet heat transfer coefficient remains constant due to the constant inflow of R410A, whereas the overall heat transfer coefficient of the outlet decreases with time. Additionally, the gas phase distribution at the outlet section of Fig. 8a and b is observed. The process of vaporization in the inner casing results in an increase of vaporization core and accumulation of bubbles on the annulus side. The fluid on the tube wall evaporates, creating direct contact between the tube wall and gas phase. This leads to a reduction in heat transfer coefficient due to higher heat transfer resistance. Under the 0.2 Hz sway condition, there is no change in the heat transfer coefficient at the inlet, indicating minimal sway effect on the refrigerant's heat transfer coefficient at the inlet. However, in comparison to the steady condition, the heat transfer coefficient at the outlet fluctuates more over time under the sway condition, resulting in a lower average heat transfer coefficient. Due to the intensified vaporization at the outlet of the casing annulus and the accumulation of bubbles, the working fluid at the outlet presents a gas–liquid two-phase state. The swaying condition makes the flow state of the working fluid at the outlet more complicated, while the inlet only changes the speed of the working fluid at the annulus side, which has little effect on its flow phase state. Therefore, the sway condition has a greater impact on the heat transfer coefficient of the working fluid outside the tube at the outlet than at the inlet.

### The influence of different sway frequencies on the heat transfer coefficient

The external heat transfer coefficient variation of the horizontal casing at different sway frequencies is crucial for the development of high-efficiency tube heat exchangers for offshore facilities. Thus, this study aims to investigate the sway frequency's impact on heat transfer coefficient concerning various mass flow rates and heat flux.

#### The heat transfer coefficient varies with the sway frequency at different mass flow rates

**Figure 11:**
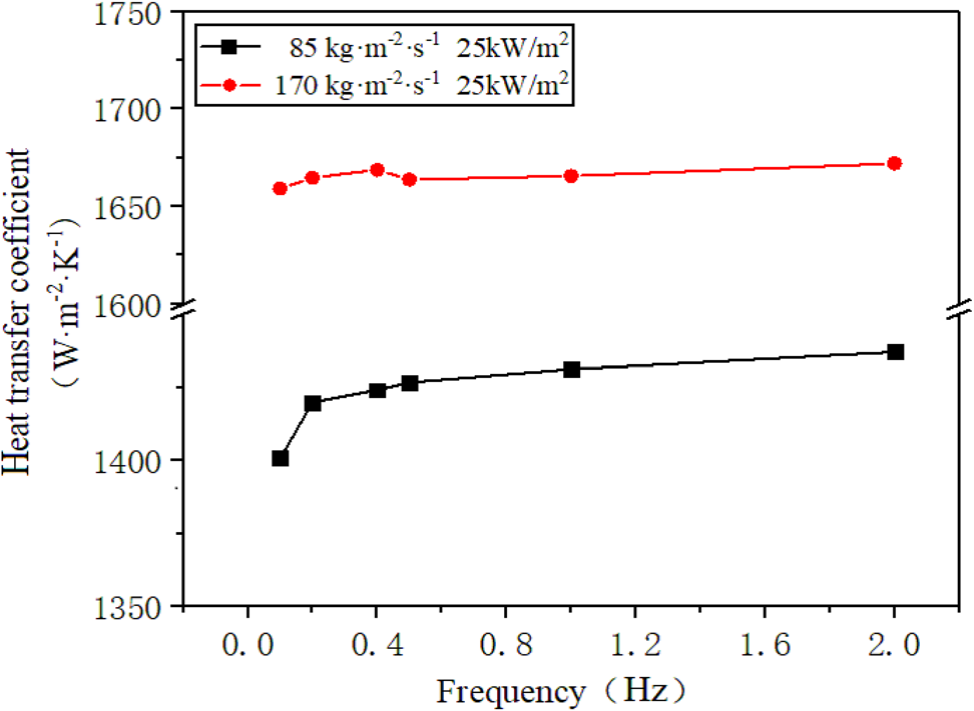
The change of heat transfer coefficient with sway frequency at different mass flow rates.

The R410A has inlet mass flow rates of 85 kg/(m^2^·s) and 170 kg/(m^2^·s), while the heat flux on the channel's inner wall measures 25 kW/m^2^ and the frequency of sway ranges from 0.1 to 2 Hz^21^. Figure 11 illustrates the alteration of the heat transfer coefficient across various mass flow rates and sway frequencies.

It is evident from Fig. 11 that an increase in mass flow rate raises the heat transfer coefficient of R410A in the annular section. This is due to the heightened inlet mass flow rate lengthening the single-phase liquid region and delaying the starting point of boiling, resulting in a reduction of the boiling region overall. With the increase in liquid mass flow rate, the rate of bubble detachment from the inner casing wall in the boiling region accelerates, thereby enhancing the disturbance effect on the fluid in the outer annulus of the horizontal tube, resulting in an increase in the convective heat transfer coefficient. At different mass flow rates, the heat transfer coefficient of R410A typically rises with the increase in the oscillation frequency. This is because of the frequent oscillations, causing the working fluid outside the tube to fluctuate significantly and hindering the formation of a dry zone in the end annulus region. This, in turn, increases the heat transfer coefficient.

#### The heat transfer coefficient varies with the sway frequency under different heat flux densities.

**Figure 12:**
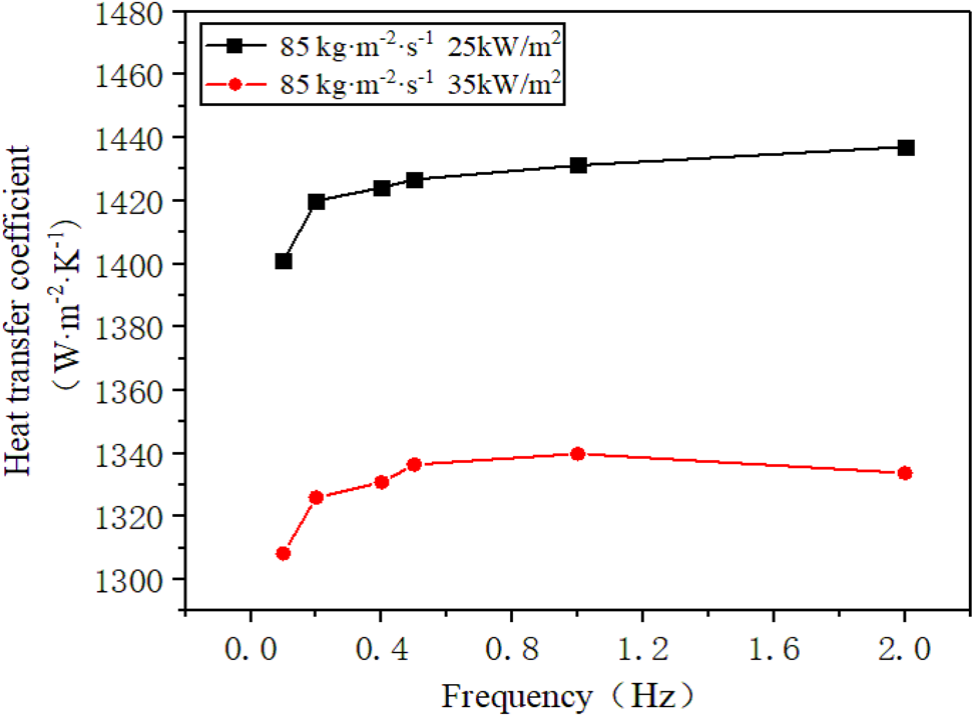
The change of heat transfer coefficient with sway frequency under different heat flux density.

The inlet mass flow rate of R410A is regulated at 85 kg/(m^2^·s), while the heat flux density on the inner wall of the flow channel is varied to 25 kW/m^2^ and 35 kW/m^2^. Figure 12 illustrates the change in the heat transfer coefficient of R410A at different heat flux densities with respect to the frequency of sway.

It is evident from Fig. 12 that the rise in heat flux density reduces the heat transfer coefficient of R410A on the annulus side. This is owing to the flow boiling heat transfer process being mainly influenced by two diverse mechanisms, namely nucleate boiling and forced convection^22^. Nucleate boiling heat transfer dominates when the working fluid has moderate and low vapor quality. The gas phase distribution of the leading and trailing segments of the casing annulus is observed. As the heat flux increases, the effect of nucleate boiling heat transfer becomes stronger, resulting in the evaporation of the working fluid on the inner wall surface of the casing. This leads to direct contact between the wall and the gas phase, causing an increase in heat transfer resistance and a decrease in the heat transfer coefficient (“Supplementary Information”).

Similarly, under varying heat fluxes, the heat transfer coefficient of R410A usually rises with an increase in sway frequency. However, at a heat flux of 35 kW/m^2^, the rise in heat transfer coefficient under high frequency sway conditions is not as noticeable as that at 25 kW/m^2^. This is because the high heat flux density causes the working fluid near the inner wall of the channel to evaporate, and the impact of lateral movement on the flow of the working fluid is not evident. When the frequency of sway increases, the heat transfer coefficient of the working fluid in the annular area outside the tube improves for different mass flow rates and heat flux densities. This is due to the high-frequency swaying, the working fluid outside the tube fluctuates violently, and it is not easy to form a dry-up zone on the end annulus side, which increases the heat transfer coefficient. Nonetheless, when subjected to high heat flux, the working fluid evaporates, causing the heat transfer coefficient to increase at a lower rate than under low heat flux conditions.

### The influence of different sway amplitude on heat transfer coefficient

**Figure 13:**
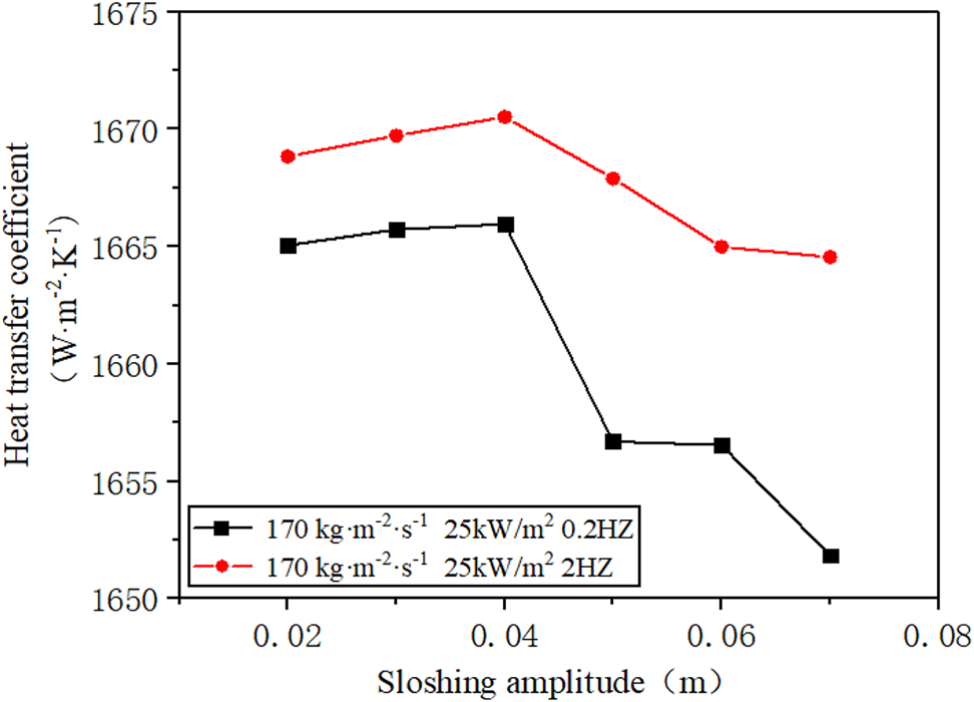
The change of heat transfer coefficient under different sway amplitude.

When the inlet mass flow rate of R410A is 170 kg/(m^2^·s), and the inner wall of the flow channel's heat flux density is 25 kW/m^2^, the sway frequency is 0.2 Hz and 2 Hz, and the sway amplitude is between 0.02 m and 0.07 m^21^, this study examines how the heat transfer coefficient is affected by changes in the sway amplitude. As shown in Fig. 13, a general decrease in the heat transfer coefficient of the working medium is observed with an increase in the sway amplitude. When the amplitude range is restricted between 0.02 m and 0.04 m, subjective evaluations of the change in heat transfer coefficient are eliminated, and the coefficient decreases markedly as amplitude increases. At high frequencies, the reduction in heat transfer coefficient is less pronounced than at low frequencies. This phenomenon results from the vigorous flow of the working fluid under high-frequency swaying, making it difficult to form a dry-out zone and resulting in a more stable heat transfer effect than low-frequency conditions. Under the low frequency condition, the amplification of the swaying amplitude makes the working fluid continuously swing to the yaw direction, which makes the dry area appear on the inner wall surface, resulting in the decrease of the heat transfer coefficient of the working fluid outside the tube. Moreover, under high frequency swaying, the working fluid experiences violent flow, making it difficult to form a dry-up zone. Consequently, the heat transfer effect's decline tendency is lower than that observed during low frequency conditions.

## Conclusion

In order to study the flow boiling heat transfer characteristics of R410A outside the horizontal tube under rolling conditions, the flow boiling heat transfer coefficient outside the horizontal tube under stable conditions was measured and calculated by using the experimental platform. Then, the effects of different mass flow rates and heat flux densities under stable conditions are analyzed. Secondly, the numerical simulation method models the flow boiling heat transfer process of R410A in the annular region spanning 1000 mm. The fluctuations of the heat transfer coefficient with swaying frequency under distinct mass flow rates and heat fluxes in both steady and ocean conditions are examined and assessed. At the same time, the influence of different swing amplitude on the flow boiling heat transfer coefficient outside the horizontal tube is analyzed, and the following conclusions are drawn :

1. Swaying conditions reduce the heat transfer coefficient of double-pipe heat exchanger. The effect of the frequency of the swaying is greater than the effect of the amplitude of the swaying.
2. When the double-pipe heat exchanger is in the swaying condition, the heat transfer coefficient of the double-pipe heat exchanger increases as the swaying frequency increases from 0.2 to 2 Hz. However, the heat transfer coefficient of the double-pipe heat exchanger decreases as the amplitude of the swaying increases from 0.02 to 0.07 m.
3. When designing heat transfer equipment for ocean conditions, it is important to consider the effects of ocean sway on its heat transfer performance. In the future, we will explore the effects of more forms of shaking on the heat transfer performance of double-pipe heat exchanger.

### Supplementary Information

Supplementary Information.

## Data Availability

The datasets used and/or analysed during the current study available from the corresponding author on reasonable request.

## List of symbols

*G*: Mass velocity [kg·m^−^^2^·s^−^^1^]
*x*: Vapor quality
*Q*_*L*_: Heat leakage [W]
*h'*: Free convection heat transfer coefficient [W·m^−^^2^·K^−^^1^]
*A'*: The outer surface area of the experimental section [m^2^]
*T*_*w*_: The outer wall temperature of experimental section [°C]
*T*_*air*_: Ambient temperature [°C]
*Q*_*1*_: Heating on the heating rod [W]
*q*: The heat flux density of the heating rod [kW·m^−^^2^]
*r*: Radius of heating rod [m]
*L*: Length of experimental section [m]
*d*: The length from the entrance of the experimental section [m]
*h*: Heat transfer coefficient [W·m^−^^2^·K^−^^1^]
*E'*: Enhancement factor
*S'*: Suppression factor
*Bo*: Boiling number
*P*_*r*_: Specific pressure
*X*_*tt*_: Martinelli parameter.
*k*: Thermal conductivity [W·m^−^^1^·K^−^^1^]
*d*_*h*_: Equivalent diameter [m]
*M*: Molecular weight
*T*_*sat*_: Saturation temperature [°C]
*Re*: Reynolds number
*Pr*: Prandtl number
*G*_*k*_: Turbulent kinetic energy generated by laminar velocity gradient [J]
*G*_*b*_: Turbulent kinetic energy generated by buoyancy [J]
*Y*_*M*_: Wave caused by turbulent diffusion
*E*: Specific internal energy [J]
*E*_*q*_: Energy of the qth phase [J]
*C*_*p,q*_: Specific heat capacity at constant pressure of liquid phase [J·kg^−^^1^·K^−^^1^]
*p*: Pressure [N]
*g*: Gravity acceleration vector [m·s^−^^2^]
*v*: Base velocity of inlet working fluid [m·s^−^^1^]
*A*: Sloshing amplitude [m]
*f*: Sloshing frequency [Hz]
*S*_*E*_: Energy source [J/m^3^·s]

## Greek symbols

*μ*: Dynamic viscosity [Pa s^−^^1^]
*σ*: Surface tension [N m^−^^1^] ; black body radiation constant [W/(m^2^·K^4^)]
*α*: Phase volume fraction
*ρ*: Density [kg m^−^^3^]
*λ*: Latent heat [J kg^−^^1^] ; thermal conductivity [W·m^−^^1^·K^−^^1^]

## Subscripts

*g*: Gas phase
*l*: Liquid phase
*nb*: Nucleate boiling
*cb*: Convective boiling
*vol*: Volume
