# Supplementary material for: Study on boiling heat transfer characteristics of R410A outside horizontal tube under swaying condition
Source: Sci Rep. 2024 Jan 23;14:1979. doi: 10.1038/s41598-024-52568-5 (PMC11294476; doi:10.1038/s41598-024-52568-5)
Supplement: Supplementary file 1 — Supplementary Information. [file 41598_2024_52568_MOESM1_ESM.docx]

Database


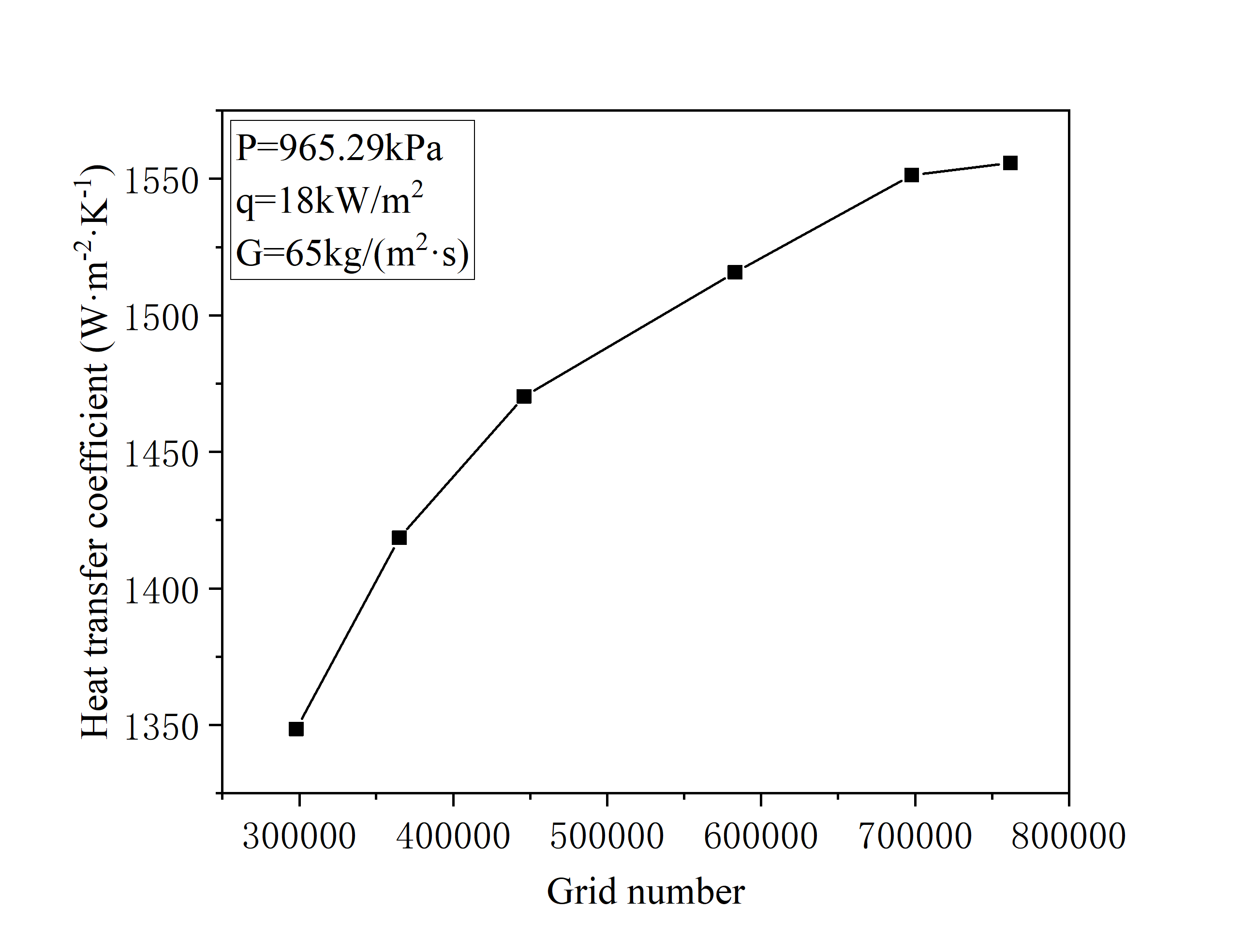


Fig. 4. Grid independence verification

| Grid number | Heat transfer coefficient(W·m^-2^·K^-1^) |
| --- | --- |
| 298000 | 1348.5 |
| 365000 | 1418.52 |
| 446000 | 1470.27 |
| 583000 | 1515.75 |
| 697820 | 1551.33 |
| 762000 | 1555.81 |


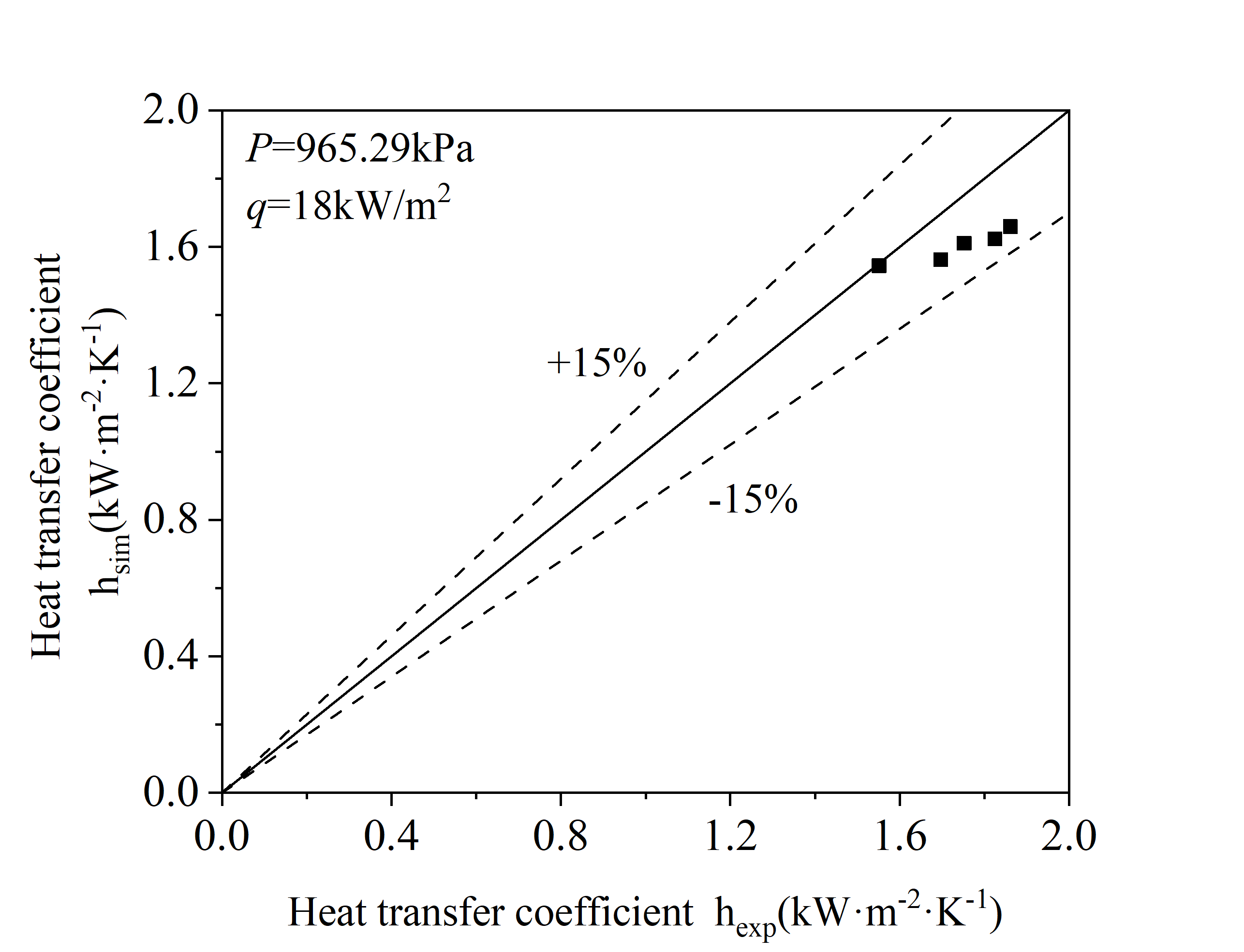


| Heat transfer coefficient hexp (kW·m^-2^·K^-1^) | Heat transfer coefficient hsim (kW·m^-2^·K^-1^) |
| --- | --- |
| 1.551 | 1.54417 |
| 1.697 | 1.56182 |
| 1.752 | 1.61081 |
| 1.825 | 1.62347 |
| 1.861 | 1.65905 |


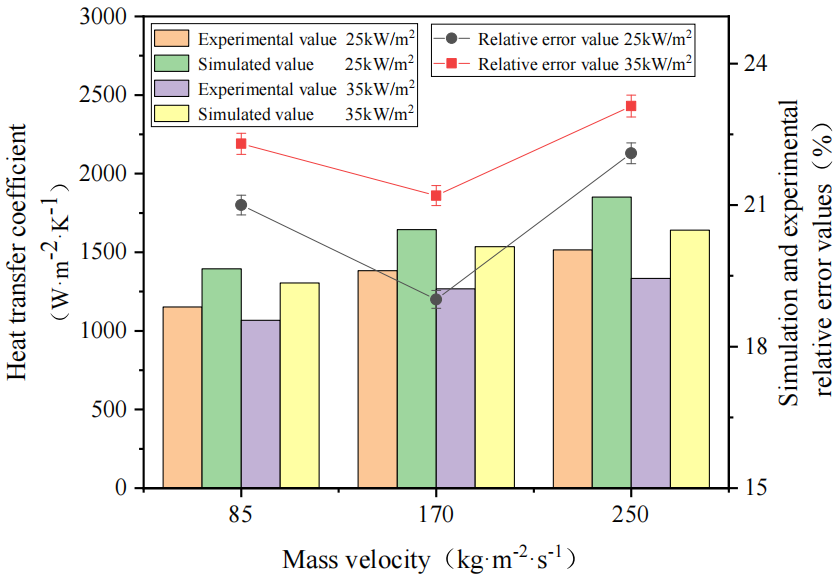


Fig 6 Comparison of experimental and simulated values of heat transfer coefficient under stable conditions

| Mass velocity（kg·m-2·s-1） | Experimental value 25Kw/m^2^ | Simulated value 25Kw/m^2^ | Experimental value 35Kw/m^2^ | Simulated value 35Kw/m^2^ | Simulation and experimental  relative error values  25Kw/m^2^ | Simulation and experimental  relative error values  35Kw/m^2^ |
| --- | --- | --- | --- | --- | --- | --- |
| 85 | 1152.9 | 1395 | 1067.1 | 1305 | 21 | 22.3 |
| 170 | 1382.4 | 1645 | 1267.3 | 1536 | 19 | 21.2 |
| 250 | 1515.9 | 1851 | 1333.1 | 1641 | 22.1 | 23.1 |


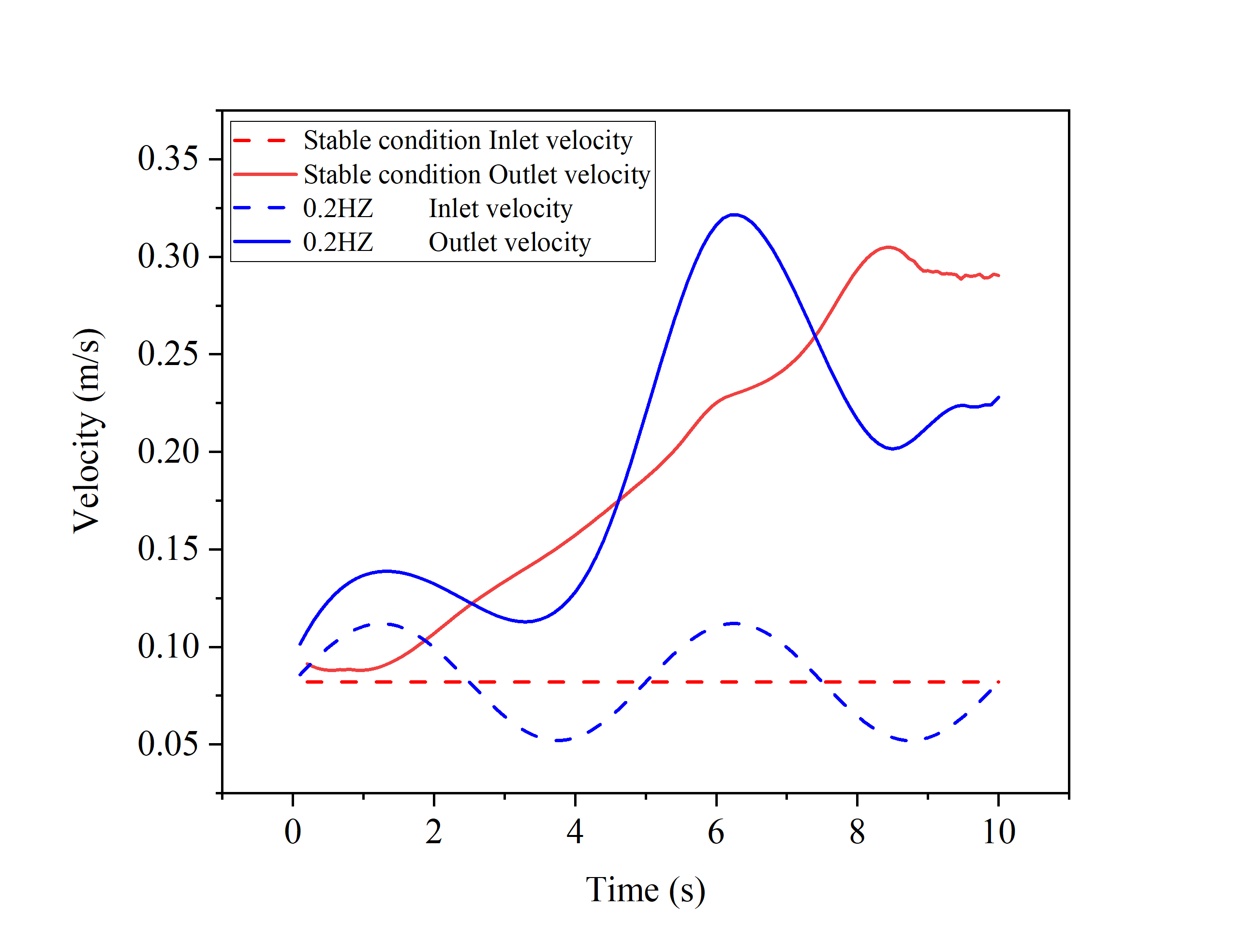


Fig. 8. The variation of inlet and outlet velocity with time

| Time(s) | Inlet velocity (m/s) | Outlet velocity (m/s) | Time(s) | Inlet velocity (m/s) | Outlet velocity (m/s) |
| --- | --- | --- | --- | --- | --- |
| Stable condition | Stable condition | Stable condition | 0.2HZ | 0.2HZ | 0.2HZ |
| 0.2 | 0.082 | 0.09132 | 0.1 | 0.08576 | 0.10151 |
| 0.26667 | 0.082 | 0.09015 | 0.2 | 0.08946 | 0.10804 |
| 0.33333 | 0.082 | 0.08925 | 0.3 | 0.09304 | 0.11384 |
| 0.4 | 0.082 | 0.08861 | 0.4 | 0.09645 | 0.11894 |
| 0.46667 | 0.082 | 0.0882 | 0.5 | 0.09963 | 0.12337 |
| 0.53333 | 0.082 | 0.088 | 0.6 | 0.10253 | 0.12717 |
| 0.6 | 0.082 | 0.08811 | 0.7 | 0.10511 | 0.13037 |
| 0.66667 | 0.082 | 0.08835 | 0.8 | 0.10732 | 0.133 |
| 0.73333 | 0.082 | 0.08825 | 0.9 | 0.10914 | 0.1351 |
| 0.8 | 0.082 | 0.08861 | 1 | 0.11053 | 0.13669 |
| 0.86667 | 0.082 | 0.0882 | 1.1 | 0.11146 | 0.13782 |
| 0.93333 | 0.082 | 0.08802 | 1.2 | 0.11194 | 0.13851 |
| 1 | 0.082 | 0.08807 | 1.3 | 0.11194 | 0.13879 |
| 1.06667 | 0.082 | 0.08832 | 1.4 | 0.11147 | 0.13871 |
| 1.13333 | 0.082 | 0.08876 | 1.5 | 0.11054 | 0.13829 |
| 1.2 | 0.082 | 0.08939 | 1.6 | 0.10916 | 0.13757 |
| 1.26667 | 0.082 | 0.09019 | 1.7 | 0.10735 | 0.13658 |
| 1.33333 | 0.082 | 0.09115 | 1.8 | 0.10514 | 0.13535 |
| 1.4 | 0.082 | 0.09226 | 1.9 | 0.10256 | 0.13392 |
| 1.46667 | 0.082 | 0.0935 | 2 | 0.09966 | 0.13232 |
| 1.53333 | 0.082 | 0.09487 | 2.1 | 0.09649 | 0.13057 |
| 1.6 | 0.082 | 0.09634 | 2.2 | 0.09308 | 0.12873 |
| 1.66667 | 0.082 | 0.09792 | 2.3 | 0.0895 | 0.12681 |
| 1.73333 | 0.082 | 0.09959 | 2.4 | 0.08581 | 0.12485 |
| 1.8 | 0.082 | 0.10133 | 2.5 | 0.08205 | 0.12289 |
| 1.86667 | 0.082 | 0.10314 | 2.6 | 0.07829 | 0.12096 |
| 1.93333 | 0.082 | 0.105 | 2.7 | 0.07459 | 0.1191 |
| 2 | 0.082 | 0.1069 | 2.8 | 0.07101 | 0.11739 |
| 2.06667 | 0.082 | 0.10884 | 2.9 | 0.0676 | 0.11586 |
| 2.13333 | 0.082 | 0.11079 | 3 | 0.06441 | 0.11459 |
| 2.2 | 0.082 | 0.11274 | 3.1 | 0.06151 | 0.11363 |
| 2.26667 | 0.082 | 0.11469 | 3.2 | 0.05892 | 0.11303 |
| 2.33333 | 0.082 | 0.11662 | 3.3 | 0.0567 | 0.11286 |
| 2.4 | 0.082 | 0.11852 | 3.4 | 0.05488 | 0.11317 |
| 2.46667 | 0.082 | 0.12038 | 3.5 | 0.05349 | 0.11402 |
| 2.53333 | 0.082 | 0.12219 | 3.6 | 0.05254 | 0.11546 |
| 2.6 | 0.082 | 0.12394 | 3.7 | 0.05206 | 0.11754 |
| 2.66667 | 0.082 | 0.12563 | 3.8 | 0.05205 | 0.12031 |
| 2.73333 | 0.082 | 0.12729 | 3.9 | 0.05252 | 0.12383 |
| 2.8 | 0.082 | 0.1289 | 4 | 0.05344 | 0.12814 |
| 2.86667 | 0.082 | 0.13048 | 4.1 | 0.05482 | 0.13329 |
| 2.93333 | 0.082 | 0.13204 | 4.2 | 0.05663 | 0.13934 |
| 3 | 0.082 | 0.13356 | 4.3 | 0.05883 | 0.14634 |
| 3.06667 | 0.082 | 0.13507 | 4.4 | 0.0614 | 0.15433 |
| 3.13333 | 0.082 | 0.13657 | 4.5 | 0.0643 | 0.16335 |
| 3.2 | 0.082 | 0.13806 | 4.6 | 0.06747 | 0.17335 |
| 3.26667 | 0.082 | 0.13956 | 4.7 | 0.07087 | 0.18413 |
| 3.33333 | 0.082 | 0.14105 | 4.8 | 0.07445 | 0.19554 |
| 3.4 | 0.082 | 0.14256 | 4.9 | 0.07815 | 0.20738 |
| 3.46667 | 0.082 | 0.14407 | 5 | 0.0819 | 0.21949 |
| 3.53333 | 0.082 | 0.14562 | 5.1 | 0.08566 | 0.23169 |
| 3.6 | 0.082 | 0.14718 | 5.2 | 0.08936 | 0.2438 |
| 3.66667 | 0.082 | 0.14878 | 5.3 | 0.09295 | 0.25565 |
| 3.73333 | 0.082 | 0.15042 | 5.4 | 0.09636 | 0.26706 |
| 3.8 | 0.082 | 0.15209 | 5.5 | 0.09955 | 0.27785 |
| 3.86667 | 0.082 | 0.15381 | 5.6 | 0.10246 | 0.28785 |
| 3.93333 | 0.082 | 0.15556 | 5.7 | 0.10505 | 0.29688 |
| 4 | 0.082 | 0.15734 | 5.8 | 0.10727 | 0.30477 |
| 4.06667 | 0.082 | 0.15915 | 5.9 | 0.1091 | 0.31134 |
| 4.13333 | 0.082 | 0.161 | 6 | 0.1105 | 0.31642 |
| 4.2 | 0.082 | 0.16287 | 6.1 | 0.11145 | 0.31982 |
| 4.26667 | 0.082 | 0.16477 | 6.2 | 0.11193 | 0.32152 |
| 4.33333 | 0.082 | 0.16668 | 6.3 | 0.11195 | 0.32164 |
| 4.4 | 0.082 | 0.16862 | 6.4 | 0.11149 | 0.32033 |
| 4.46667 | 0.082 | 0.17058 | 6.5 | 0.11057 | 0.31773 |
| 4.53333 | 0.082 | 0.17255 | 6.6 | 0.1092 | 0.31399 |
| 4.6 | 0.082 | 0.17454 | 6.7 | 0.1074 | 0.30925 |
| 4.66667 | 0.082 | 0.17653 | 6.8 | 0.1052 | 0.30365 |
| 4.73333 | 0.082 | 0.17854 | 6.9 | 0.10263 | 0.29734 |
| 4.8 | 0.082 | 0.18055 | 7 | 0.09974 | 0.29043 |
| 4.86667 | 0.082 | 0.18257 | 7.1 | 0.09657 | 0.28306 |
| 4.93333 | 0.082 | 0.18459 | 7.2 | 0.09317 | 0.27535 |
| 5 | 0.082 | 0.18663 | 7.3 | 0.0896 | 0.26742 |
| 5.06667 | 0.082 | 0.18872 | 7.4 | 0.0859 | 0.25941 |
| 5.13333 | 0.082 | 0.19087 | 7.5 | 0.08214 | 0.25143 |
| 5.2 | 0.082 | 0.1931 | 7.6 | 0.07838 | 0.24362 |
| 5.26667 | 0.082 | 0.19544 | 7.7 | 0.07468 | 0.2361 |
| 5.33333 | 0.082 | 0.19789 | 7.8 | 0.0711 | 0.229 |
| 5.4 | 0.082 | 0.20049 | 7.9 | 0.06768 | 0.22244 |
| 5.46667 | 0.082 | 0.20325 | 8 | 0.06449 | 0.21655 |
| 5.53333 | 0.082 | 0.20617 | 8.1 | 0.06158 | 0.21145 |
| 5.6 | 0.082 | 0.2092 | 8.2 | 0.05898 | 0.20727 |
| 5.66667 | 0.082 | 0.21226 | 8.3 | 0.05676 | 0.20414 |
| 5.73333 | 0.082 | 0.21527 | 8.4 | 0.05492 | 0.20218 |
| 5.8 | 0.082 | 0.21815 | 8.5 | 0.05352 | 0.20152 |
| 5.86667 | 0.082 | 0.22082 | 8.6 | 0.05256 | 0.20218 |
| 5.93333 | 0.082 | 0.2232 | 8.7 | 0.05207 | 0.20392 |
| 6 | 0.082 | 0.2252 | 8.8 | 0.05205 | 0.20648 |
| 6.06667 | 0.082 | 0.22678 | 8.9 | 0.0525 | 0.20958 |
| 6.13333 | 0.082 | 0.22798 | 9 | 0.05342 | 0.21293 |
| 6.2 | 0.082 | 0.22895 | 9.1 | 0.05478 | 0.21626 |
| 6.26667 | 0.082 | 0.22978 | 9.2 | 0.05658 | 0.21931 |
| 6.33333 | 0.082 | 0.23061 | 9.3 | 0.05877 | 0.22178 |
| 6.4 | 0.082 | 0.23147 | 9.4 | 0.06133 | 0.22341 |
| 6.46667 | 0.082 | 0.23239 | 9.5 | 0.06422 | 0.22392 |
| 6.53333 | 0.082 | 0.23337 | 9.6 | 0.06739 | 0.22304 |
| 6.6 | 0.082 | 0.23443 | 9.7 | 0.07078 | 0.223 |
| 6.66667 | 0.082 | 0.2356 | 9.8 | 0.07436 | 0.224 |
| 6.73333 | 0.082 | 0.23687 | 9.9 | 0.07805 | 0.2242 |
| 6.8 | 0.082 | 0.23828 | 10 | 0.08181 | 0.228 |
| 6.86667 | 0.082 | 0.23982 |  |  |  |
| 6.93333 | 0.082 | 0.24153 |  |  |  |
| 7 | 0.082 | 0.24341 |  |  |  |
| 7.06667 | 0.082 | 0.24548 |  |  |  |
| 7.13333 | 0.082 | 0.24775 |  |  |  |
| 7.2 | 0.082 | 0.25025 |  |  |  |
| 7.26667 | 0.082 | 0.25298 |  |  |  |
| 7.33333 | 0.082 | 0.25596 |  |  |  |
| 7.4 | 0.082 | 0.2592 |  |  |  |
| 7.46667 | 0.082 | 0.26272 |  |  |  |
| 7.53333 | 0.082 | 0.26649 |  |  |  |
| 7.6 | 0.082 | 0.27043 |  |  |  |
| 7.66667 | 0.082 | 0.27447 |  |  |  |
| 7.73333 | 0.082 | 0.27853 |  |  |  |
| 7.8 | 0.082 | 0.28254 |  |  |  |
| 7.86667 | 0.082 | 0.28641 |  |  |  |
| 7.93333 | 0.082 | 0.29008 |  |  |  |
| 8 | 0.082 | 0.29346 |  |  |  |
| 8.06667 | 0.082 | 0.29649 |  |  |  |
| 8.13333 | 0.082 | 0.2991 |  |  |  |
| 8.2 | 0.082 | 0.30127 |  |  |  |
| 8.26667 | 0.082 | 0.30296 |  |  |  |
| 8.33333 | 0.082 | 0.30415 |  |  |  |
| 8.4 | 0.082 | 0.30481 |  |  |  |
| 8.46667 | 0.082 | 0.30489 |  |  |  |
| 8.53333 | 0.082 | 0.30439 |  |  |  |
| 8.6 | 0.082 | 0.30326 |  |  |  |
| 8.66667 | 0.082 | 0.30148 |  |  |  |
| 8.73333 | 0.082 | 0.29901 |  |  |  |
| 8.8 | 0.082 | 0.29783 |  |  |  |
| 8.86667 | 0.082 | 0.29498 |  |  |  |
| 8.93333 | 0.082 | 0.2927 |  |  |  |
| 9 | 0.082 | 0.2929 |  |  |  |
| 9.06667 | 0.082 | 0.29225 |  |  |  |
| 9.13333 | 0.082 | 0.2926 |  |  |  |
| 9.2 | 0.082 | 0.29124 |  |  |  |
| 9.26667 | 0.082 | 0.29137 |  |  |  |
| 9.33333 | 0.082 | 0.29117 |  |  |  |
| 9.4 | 0.082 | 0.29084 |  |  |  |
| 9.46667 | 0.082 | 0.28858 |  |  |  |
| 9.53333 | 0.082 | 0.29059 |  |  |  |
| 9.6 | 0.082 | 0.29006 |  |  |  |
| 9.66667 | 0.082 | 0.29018 |  |  |  |
| 9.73333 | 0.082 | 0.29115 |  |  |  |
| 9.8 | 0.082 | 0.28917 |  |  |  |
| 9.86667 | 0.082 | 0.28942 |  |  |  |
| 9.93333 | 0.082 | 0.29111 |  |  |  |
| 10 | 0.082 | 0.29043 |  |  |  |


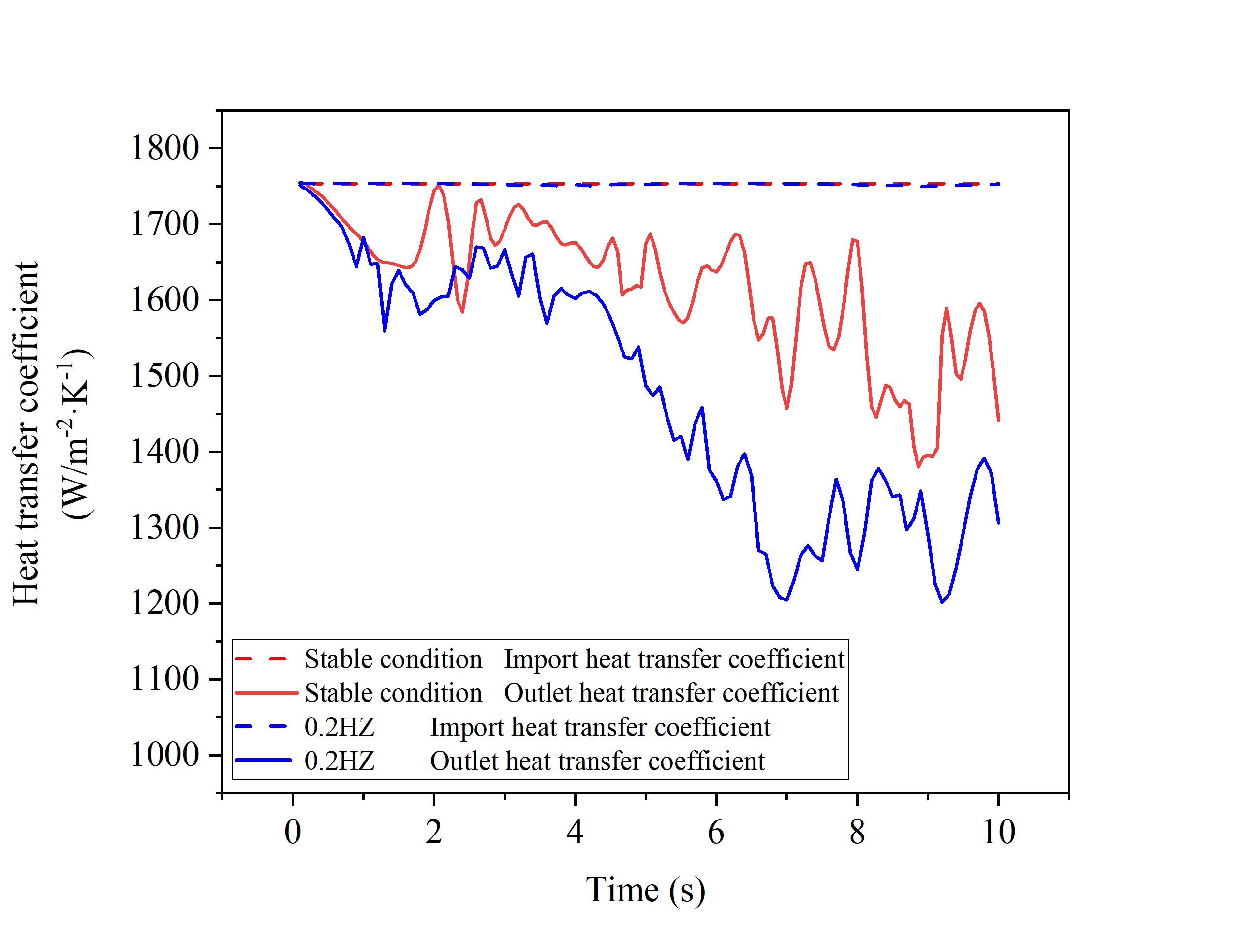


Fig. 9. Variation of heat transfer coefficient of inlet and outlet with time

| Time(s) | Import heat transfer coefficient  (W·m^-2^·K^-1^) | Outlet heat transfer coefficient  (W·m^-2^·K^-1^) | Time(s) | Import heat transfer coefficient  (W·m^-2^·K^-1^) | Outlet heat transfer coefficient  (W·m^-2^·K^-1^) |
| --- | --- | --- | --- | --- | --- |
| Stable condition | Stable condition | Stable condition | 0.2HZ | 0.2HZ | 0.2HZ |
| 0.2 | 1753.88562 | 1751.13892 | 0.1 | 1754.54846 | 1751.15877 |
| 0.26667 | 1753.73218 | 1747.04059 | 0.2 | 1753.73767 | 1745.59285 |
| 0.33333 | 1753.60144 | 1742.64455 | 0.3 | 1753.40393 | 1737.90171 |
| 0.4 | 1753.51611 | 1737.65308 | 0.4 | 1753.50195 | 1728.68848 |
| 0.46667 | 1753.4902 | 1731.84412 | 0.5 | 1753.6051 | 1718.38212 |
| 0.53333 | 1753.50298 | 1725.29836 | 0.6 | 1753.68506 | 1706.56234 |
| 0.6 | 1753.52502 | 1718.17212 | 0.7 | 1753.75024 | 1695.55092 |
| 0.66667 | 1753.53383 | 1710.6788 | 0.8 | 1753.79346 | 1672.98476 |
| 0.73333 | 1753.53456 | 1703.25998 | 0.9 | 1753.83289 | 1644.09506 |
| 0.8 | 1753.53931 | 1696.41431 | 1 | 1753.89734 | 1682.77536 |
| 0.86667 | 1753.55572 | 1690.38423 | 1.1 | 1753.93274 | 1647.55108 |
| 0.93333 | 1753.57374 | 1684.38746 | 1.2 | 1753.93835 | 1648.17212 |
| 1 | 1753.57886 | 1677.3855 | 1.3 | 1753.89307 | 1559.46354 |
| 1.06667 | 1753.56152 | 1668.84666 | 1.4 | 1753.86499 | 1620.69118 |
| 1.13333 | 1753.53194 | 1660.2664 | 1.5 | 1753.89136 | 1639.69852 |
| 1.2 | 1753.50525 | 1653.64697 | 1.6 | 1753.89221 | 1620.14905 |
| 1.26667 | 1753.49382 | 1650.35586 | 1.7 | 1753.84644 | 1609.87067 |
| 1.33333 | 1753.49886 | 1649.22151 | 1.8 | 1753.75256 | 1581.34555 |
| 1.4 | 1753.5188 | 1648.43762 | 1.9 | 1753.68787 | 1587.63036 |
| 1.46667 | 1753.54915 | 1646.67694 | 2 | 1753.65772 | 1599.65735 |
| 1.53333 | 1753.57371 | 1644.52838 | 2.1 | 1753.61719 | 1604.22717 |
| 1.6 | 1753.57336 | 1643.05994 | 2.2 | 1753.54492 | 1605.36669 |
| 1.66667 | 1753.53753 | 1643.78978 | 2.3 | 1753.45227 | 1644.14814 |
| 1.73333 | 1753.4897 | 1650.03691 | 2.4 | 1753.30774 | 1640.07239 |
| 1.8 | 1753.46191 | 1665.57056 | 2.5 | 1753.21069 | 1628.97768 |
| 1.86667 | 1753.47608 | 1691.88984 | 2.6 | 1753.08765 | 1670.28327 |
| 1.93333 | 1753.51366 | 1721.41343 | 2.7 | 1752.76221 | 1668.6516 |
| 2 | 1753.54602 | 1744.28992 | 2.8 | 1752.42749 | 1642.18623 |
| 2.06667 | 1753.5521 | 1751.80966 | 2.9 | 1752.37939 | 1645.07819 |
| 2.13333 | 1753.54128 | 1739.8302 | 3 | 1752.11291 | 1666.91022 |
| 2.2 | 1753.53052 | 1705.35083 | 3.1 | 1751.89648 | 1633.82401 |
| 2.26667 | 1753.5328 | 1650.83644 | 3.2 | 1751.41638 | 1605.24686 |
| 2.33333 | 1753.54511 | 1600.61413 | 3.3 | 1751.93201 | 1656.74027 |
| 2.4 | 1753.56042 | 1584.47656 | 3.4 | 1752.06689 | 1660.87121 |
| 2.46667 | 1753.57225 | 1620.73482 | 3.5 | 1751.4596 | 1603.16361 |
| 2.53333 | 1753.5761 | 1681.77398 | 3.6 | 1751.87988 | 1568.58646 |
| 2.6 | 1753.56799 | 1728.49768 | 3.7 | 1751.22937 | 1605.63317 |
| 2.66667 | 1753.54673 | 1732.55329 | 3.8 | 1750.77734 | 1615.39707 |
| 2.73333 | 1753.52218 | 1708.56314 | 3.9 | 1751.36511 | 1606.81092 |
| 2.8 | 1753.50696 | 1681.89343 | 4 | 1752.17664 | 1602.26994 |
| 2.86667 | 1753.50995 | 1672.49537 | 4.1 | 1751.42822 | 1609.28829 |
| 2.93333 | 1753.52501 | 1678.66023 | 4.2 | 1750.94824 | 1611.48658 |
| 3 | 1753.54224 | 1693.26428 | 4.3 | 1750.98315 | 1606.59483 |
| 3.06667 | 1753.5535 | 1709.56163 | 4.4 | 1751.69568 | 1594.78799 |
| 3.13333 | 1753.55777 | 1722.31762 | 4.5 | 1752.24329 | 1576.27102 |
| 3.2 | 1753.55579 | 1726.67541 | 4.6 | 1752.38696 | 1551.22879 |
| 3.26667 | 1753.54858 | 1719.94506 | 4.7 | 1752.62146 | 1525.16924 |
| 3.33333 | 1753.53834 | 1708.10423 | 4.8 | 1752.86963 | 1522.87322 |
| 3.4 | 1753.52759 | 1699.29749 | 4.9 | 1752.90247 | 1538.34359 |
| 3.46667 | 1753.51897 | 1699.10064 | 5 | 1752.80945 | 1487.63418 |
| 3.53333 | 1753.51578 | 1702.8145 | 5.1 | 1752.96777 | 1473.72554 |
| 3.6 | 1753.52148 | 1703.17114 | 5.2 | 1753.25464 | 1485.54574 |
| 3.66667 | 1753.53693 | 1695.23706 | 5.3 | 1753.42481 | 1446.68693 |
| 3.73333 | 1753.55251 | 1683.41649 | 5.4 | 1753.52588 | 1415.05247 |
| 3.8 | 1753.55603 | 1674.44812 | 5.5 | 1753.61401 | 1420.83329 |
| 3.86667 | 1753.54004 | 1672.98649 | 5.6 | 1753.67212 | 1389.67786 |
| 3.93333 | 1753.51615 | 1675.34953 | 5.7 | 1753.73755 | 1437.60732 |
| 4 | 1753.50073 | 1675.771 | 5.8 | 1753.81152 | 1459.19222 |
| 4.06667 | 1753.50538 | 1670.06667 | 5.9 | 1753.8667 | 1376.39921 |
| 4.13333 | 1753.52252 | 1660.3802 | 6 | 1753.87549 | 1362.10724 |
| 4.2 | 1753.53979 | 1650.43726 | 6.1 | 1753.87903 | 1337.43006 |
| 4.26667 | 1753.54776 | 1643.85235 | 6.2 | 1753.91943 | 1341.36819 |
| 4.33333 | 1753.54865 | 1643.79564 | 6.3 | 1753.927 | 1380.65005 |
| 4.4 | 1753.54761 | 1653.32617 | 6.4 | 1753.91956 | 1397.81771 |
| 4.46667 | 1753.54826 | 1671.56454 | 6.5 | 1753.9115 | 1368.89048 |
| 4.53333 | 1753.54815 | 1681.87796 | 6.6 | 1753.87378 | 1270.22821 |
| 4.6 | 1753.54334 | 1663.69531 | 6.7 | 1753.83496 | 1265.58124 |
| 4.66667 | 1753.53174 | 1606.90088 | 6.8 | 1753.81372 | 1223.78311 |
| 4.73333 | 1753.51889 | 1613.20174 | 6.9 | 1753.71985 | 1208.23307 |
| 4.8 | 1753.51221 | 1614.76062 | 7 | 1753.6012 | 1204.32585 |
| 4.86667 | 1753.51664 | 1619.35824 | 7.1 | 1753.55286 | 1231.15725 |
| 4.93333 | 1753.52719 | 1617.24799 | 7.2 | 1753.52405 | 1264.25362 |
| 5 | 1753.53638 | 1674.30164 | 7.3 | 1753.41882 | 1276.20041 |
| 5.06667 | 1753.53838 | 1687.41294 | 7.4 | 1753.30347 | 1263.27349 |
| 5.13333 | 1753.53408 | 1667.56381 | 7.5 | 1753.2146 | 1256.40326 |
| 5.2 | 1753.526 | 1636.75818 | 7.6 | 1753.01331 | 1313.75745 |
| 5.26667 | 1753.51709 | 1612.76049 | 7.7 | 1752.64392 | 1363.90246 |
| 5.33333 | 1753.5118 | 1596.3775 | 7.8 | 1752.44226 | 1334.03032 |
| 5.4 | 1753.51501 | 1584.17639 | 7.9 | 1752.4259 | 1266.68691 |
| 5.46667 | 1753.52895 | 1574.17416 | 8 | 1752.3335 | 1244.58777 |
| 5.53333 | 1753.54533 | 1570.18687 | 8.1 | 1751.9895 | 1291.54623 |
| 5.6 | 1753.55322 | 1577.48035 | 8.2 | 1751.49439 | 1362.31774 |
| 5.66667 | 1753.5455 | 1598.45668 | 8.3 | 1751.56079 | 1378.12352 |
| 5.73333 | 1753.53015 | 1624.06245 | 8.4 | 1751.42407 | 1362.04452 |
| 5.8 | 1753.51892 | 1642.38037 | 8.5 | 1751.125 | 1340.90654 |
| 5.86667 | 1753.52023 | 1645.30546 | 8.6 | 1751.67578 | 1343.19251 |
| 5.93333 | 1753.52914 | 1639.98182 | 8.7 | 1751.53442 | 1297.32715 |
| 6 | 1753.53735 | 1637.36572 | 8.8 | 1750.51782 | 1312.26277 |
| 6.06667 | 1753.53827 | 1645.51311 | 8.9 | 1749.9657 | 1348.54309 |
| 6.13333 | 1753.53201 | 1660.87842 | 9 | 1750.32324 | 1291.1806 |
| 6.2 | 1753.52039 | 1677.01575 | 9.1 | 1750.61975 | 1226.20566 |
| 6.26667 | 1753.50602 | 1687.3917 | 9.2 | 1750.88855 | 1201.56157 |
| 6.33333 | 1753.49486 | 1685.12284 | 9.3 | 1751.17883 | 1212.28098 |
| 6.4 | 1753.49365 | 1663.23828 | 9.4 | 1751.39416 | 1246.78968 |
| 6.46667 | 1753.50636 | 1619.85214 | 9.5 | 1751.89929 | 1293.55343 |
| 6.53333 | 1753.52568 | 1573.41786 | 9.6 | 1751.90686 | 1340.98803 |
| 6.6 | 1753.5415 | 1547.47376 | 9.7 | 1751.80127 | 1377.54924 |
| 6.66667 | 1753.54598 | 1556.2423 | 9.8 | 1752.61169 | 1391.67285 |
| 6.73333 | 1753.54022 | 1576.68227 | 9.9 | 1752.71558 | 1371.79462 |
| 6.8 | 1753.52759 | 1576.4364 | 10 | 1752.98084 | 1306.36035 |
| 6.86667 | 1753.51162 | 1535.34377 |  |  |  |
| 6.93333 | 1753.49653 | 1482.02803 |  |  |  |
| 7 | 1753.48669 | 1457.30896 |  |  |  |
| 7.06667 | 1753.48566 | 1489.1935 |  |  |  |
| 7.13333 | 1753.4936 | 1554.43738 |  |  |  |
| 7.2 | 1753.50989 | 1616.98352 |  |  |  |
| 7.26667 | 1753.53249 | 1648.22658 |  |  |  |
| 7.33333 | 1753.55392 | 1649.36785 |  |  |  |
| 7.4 | 1753.56531 | 1629.06055 |  |  |  |
| 7.46667 | 1753.56134 | 1596.43263 |  |  |  |
| 7.53333 | 1753.55095 | 1562.51054 |  |  |  |
| 7.6 | 1753.54663 | 1538.79541 |  |  |  |
| 7.66667 | 1753.55656 | 1534.73915 |  |  |  |
| 7.73333 | 1753.57164 | 1551.59675 |  |  |  |
| 7.8 | 1753.57849 | 1588.57385 |  |  |  |
| 7.86667 | 1753.56748 | 1639.87862 |  |  |  |
| 7.93333 | 1753.54409 | 1679.73002 |  |  |  |
| 8 | 1753.51758 | 1677.34961 |  |  |  |
| 8.06667 | 1753.49667 | 1614.91225 |  |  |  |
| 8.13333 | 1753.48792 | 1526.40597 |  |  |  |
| 8.2 | 1753.49731 | 1458.7721 |  |  |  |
| 8.26667 | 1753.52626 | 1445.5162 |  |  |  |
| 8.33333 | 1753.55769 | 1466.40081 |  |  |  |
| 8.4 | 1753.56995 | 1487.75269 |  |  |  |
| 8.46667 | 1753.54923 | 1484.85241 |  |  |  |
| 8.53333 | 1753.51326 | 1468.79611 |  |  |  |
| 8.6 | 1753.48767 | 1459.63403 |  |  |  |
| 8.66667 | 1753.49118 | 1467.5065 |  |  |  |
| 8.73333 | 1753.51496 | 1462.91459 |  |  |  |
| 8.8 | 1753.54334 | 1406.44971 |  |  |  |
| 8.86667 | 1753.56299 | 1380.31216 |  |  |  |
| 8.93333 | 1753.57026 | 1393.13783 |  |  |  |
| 9 | 1753.56384 | 1395.17151 |  |  |  |
| 9.06667 | 1753.54451 | 1393.85734 |  |  |  |
| 9.13333 | 1753.52117 | 1405.43692 |  |  |  |
| 9.2 | 1753.50476 | 1553.3512 |  |  |  |
| 9.26667 | 1753.50314 | 1589.69594 |  |  |  |
| 9.33333 | 1753.51172 | 1551.18618 |  |  |  |
| 9.4 | 1753.52283 | 1503.19177 |  |  |  |
| 9.46667 | 1753.53057 | 1496.38224 |  |  |  |
| 9.53333 | 1753.53626 | 1522.62563 |  |  |  |
| 9.6 | 1753.54297 | 1559.08911 |  |  |  |
| 9.66667 | 1753.55226 | 1586.04751 |  |  |  |
| 9.73333 | 1753.55952 | 1596.20686 |  |  |  |
| 9.8 | 1753.55859 | 1585.38074 |  |  |  |
| 9.86667 | 1753.54516 | 1551.47887 |  |  |  |
| 9.93333 | 1753.52216 | 1500.79572 |  |  |  |
| 10 | 1753.49439 | 1441.72192 |  |  |  |


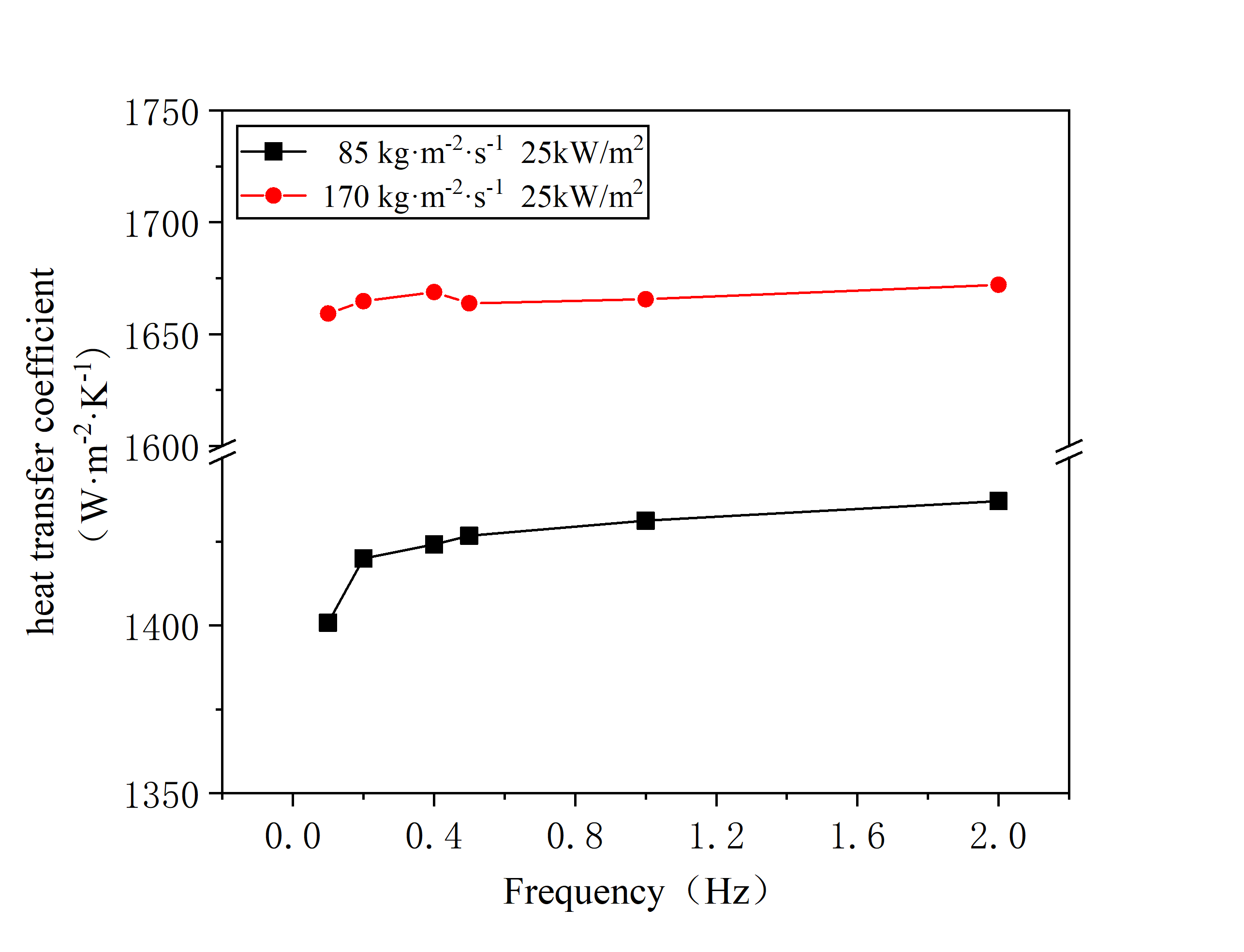


Fig. 10. The change of heat transfer coefficient with sway frequency at different mass flow rates

| Frequency(Hz) | Heat transfer coefficient  (W·m^-2^·K^-1^) | Heat transfer coefficient  (W·m^-2^·K^-1^) |
| --- | --- | --- |
|  | Mass velocity  85（kg·m-2·s-1） | Mass velocity  170（kg·m-2·s-1） |
|  | 25kW/m^2^ | 25kW/m^2^ |
| 0.1 | 1400.8575 | 1659.21 |
| 0.2 | 1419.997 | 1664.73 |
| 0.4 | 1424.1611 | 1668.8258 |
| 0.5 | 1426.8 | 1663.7878 |
| 1 | 1431.24 | 1665.6138 |
| 2 | 1437.1 | 1672.0815 |


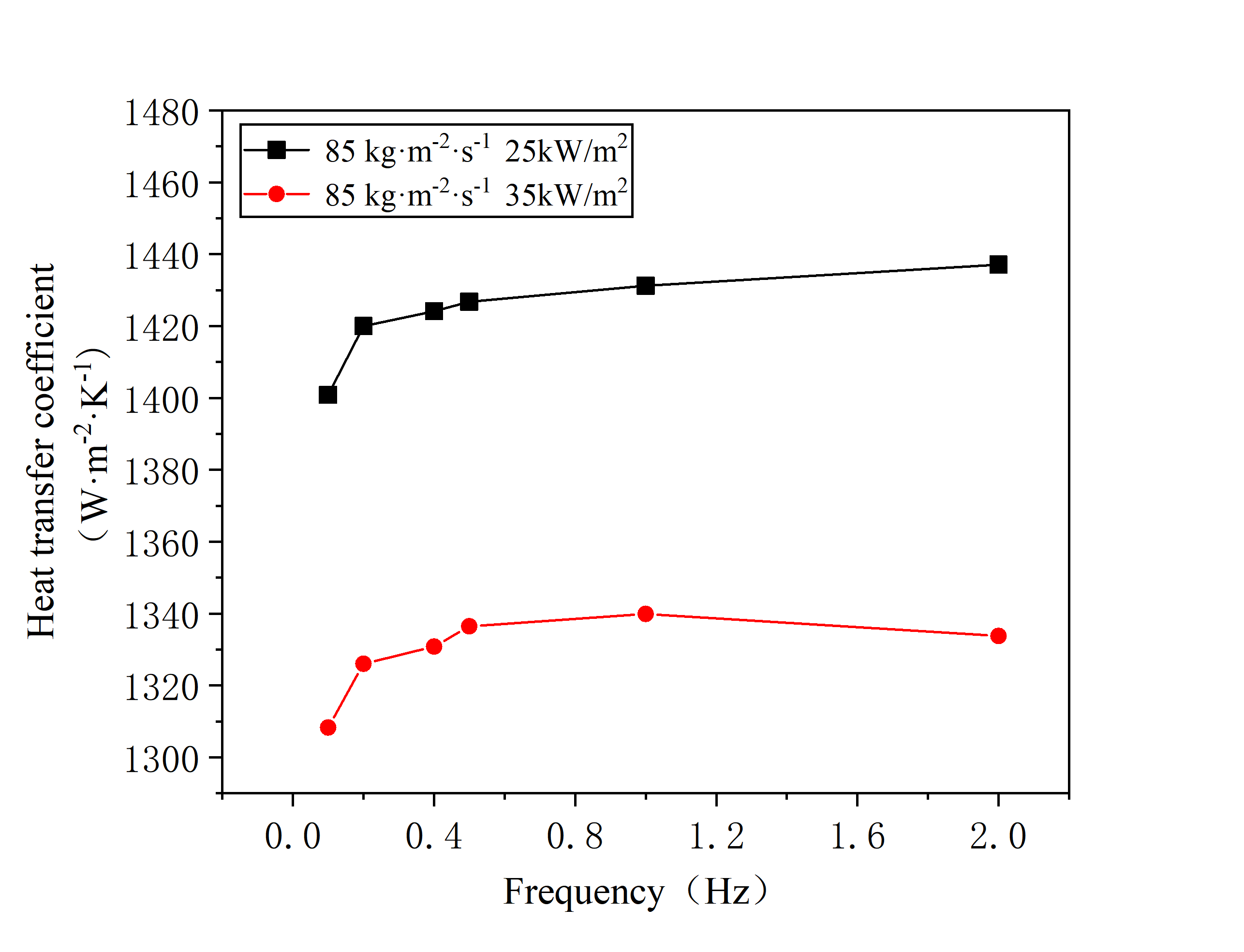


Fig. 11. The change of heat transfer coefficient with sway frequency under different heat flux density

| Frequency(Hz) | Heat transfer coefficient  (W·m^-2^·K^-1^) | Heat transfer coefficient  (W·m^-2^·K^-1^) |
| --- | --- | --- |
|  | Mass velocity  85（kg·m-2·s-1） | Mass velocity  85（kg·m-2·s-1） |
|  | 25kW/m^2^ | 35kW/m^2^ |
| 0.1 | 1400.8575 | 1308.2799 |
| 0.2 | 1419.997 | 1326.0157 |
| 0.4 | 1424.1611 | 1330.7853 |
| 0.5 | 1426.8 | 1336.445 |
| 1 | 1431.24 | 1339.869 |
| 2 | 1437.1 | 1333.7515 |


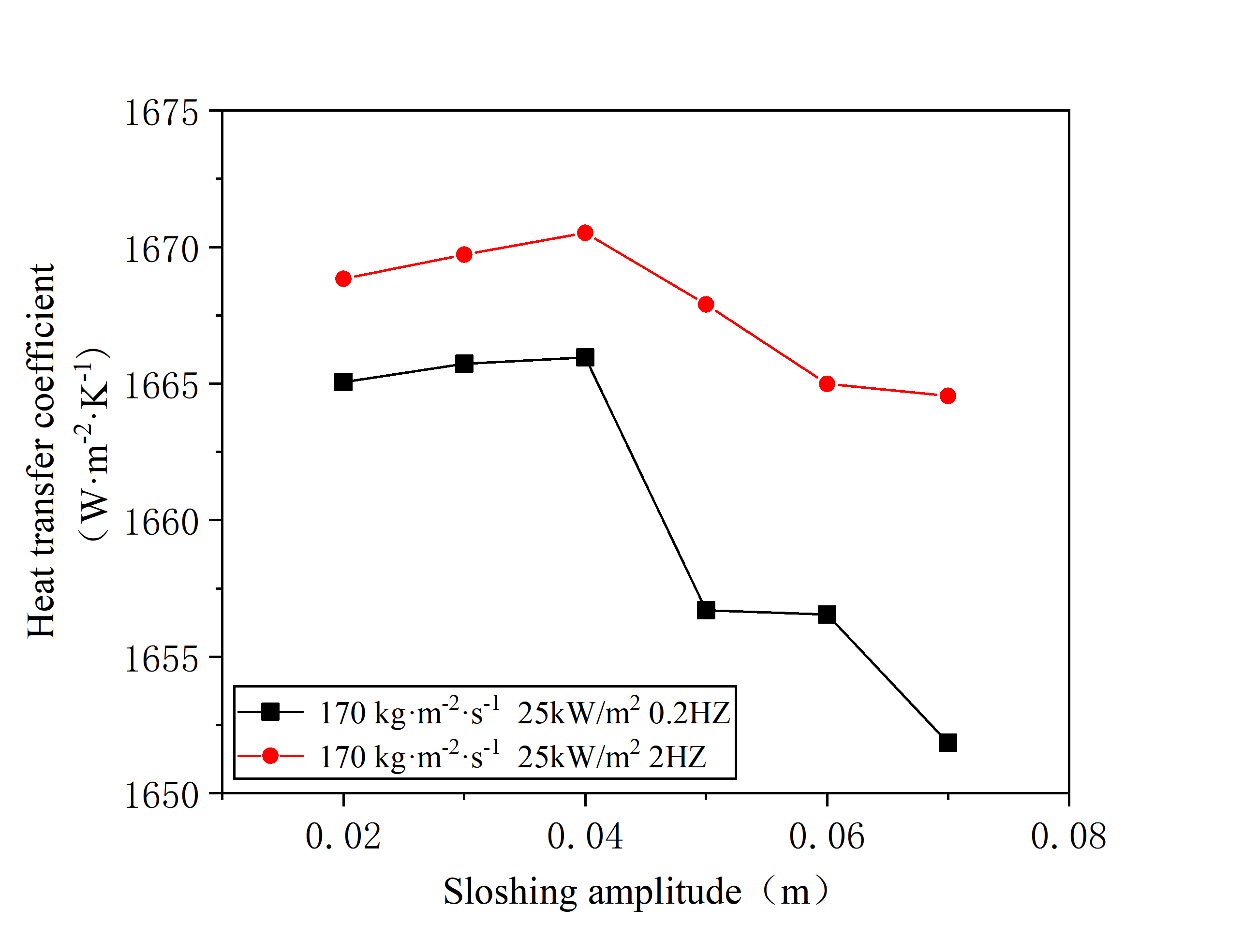


Fig. 12. The change of heat transfer coefficient under different sway amplitude

| Sloshing amplitude (m) | Heat transfer coefficient  (W·m^-2^·K^-1^) | Heat transfer coefficient  (W·m^-2^·K^-1^) |
| --- | --- | --- |
|  | Mass velocity  170（kg·m-2·s-1） | Mass velocity  170（kg·m-2·s-1） |
|  | 25kW/m^2^ | 25kW/m^2^ |
|  | 0.2Hz | 2Hz |
| 0.02 | 1665.055 | 1668.84 |
| 0.03 | 1665.726 | 1669.72 |
| 0.04 | 1665.961 | 1670.52 |
| 0.05 | 1656.699 | 1667.9 |
| 0.06 | 1656.545 | 1664.99 |
| 0.07 | 1651.843 | 1664.55 |
